# Bidirectional causal relationship between psychiatric disorders and osteoarthritis: A univariate and multivariate Mendelian randomization study

**DOI:** 10.1002/brb3.3429

**Published:** 2024-02-15

**Authors:** Jinzhi Meng, Youran Cai, Jun Yao, Haiwei Yan

**Affiliations:** ^1^ Bone and Joint Surgery The First Affiliated Hospital of Guangxi Medical University Nanning China; ^2^ Department of Ophthalmology The First Affiliated Hospital of Guangxi Medical University Nanning China; ^3^ Department of Sports Medicine The Fourth Affiliated Hospital of Guangxi Medical University Liuzhou China

**Keywords:** bipolar disorder, major depression, Mendelian randomization, osteoarthritis, schizophrenia

## Abstract

**Background:**

Observational studies have shown associations between psychiatric disorders and osteoarthritis (OA). However, the causal impact of different psychiatric disorder types on specific sites of osteoarthritis remains unclear. This study aimed to comprehensively understand the potential causal associations between psychiatric disorders and osteoarthritis using Mendelian randomization (MR) analysis.

**Methods:**

We collected data from genome‐wide association studies of knee osteoarthritis (KOA) (*n* = 403,124), hip osteoarthritis (HOA) (*n* = 393,873), osteoarthritis of the knee or hip (KHOA) (*n* = 417,596), as well as three psychiatric disorders: bipolar disorder (*n* = 41,917), major depressive disorder (*n* = 170,756), and schizophrenia (*n* = 76,755) among European populations. We applied bidirectional univariate and multivariate MR analyses, including inverse variance weighted, Mendelian randomization‐Egger, weighted median, simple mode, and weighted mode. We considered *p* < .05 as a criterion for identifying potential evidence of association. Bonferroni correction was used for multiple tests.

**Results:**

Our univariate MR analysis results demonstrated that bipolar disorder is a protective factor for KOA (OR = 0.90, 95% CI = 0.83 to 0.97, *p* = 0.0048) and may also be protective for KHOA (*p* = 0.02). Conversely, major depression has a positive causal effect on both KOA (OR = 1.27; 95% CI = 1.08 to 1.49; *p* = 0.0036) and KHOA (OR = 1.24; 95% CI = 1.12 to 1.37; *p* = 3.62×10^‐05^). Furthermore, our analysis suggested that KHOA may be a risk factor for major depression (OR = 1.06; 95% CI = 1.00 to 1.12; *p* = 0.0469) in reverse MR. After adjusting smoking (OR = 1.46; 95% CI = 1.19 to 1.65; *p* = 0.0032) and body mass index (OR = 1.44; 95% CI = 1.09 to 1.81; *p* = 8.56×10^‐04^), the casual association between major depression and KHOA remained.

**Conclusion:**

Our study indicates that major depression is a great risk factor for KHOA, increasing the likelihood of their occurrence. However, further in‐depth studies will be required to validate these results and elucidate the underlying molecular mechanisms.

## Graphical abstract

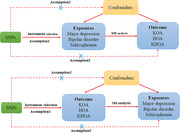

## 1 INTRODUCTION

Osteoarthritis (OA) is a degenerative disease that primarily affects articular cartilage and leads to high disability rates, particularly among middle‐aged and older individuals. Presently, it stands as the fourth most disabling disease worldwide and significantly impacts the daily lives of patients (Li et al., 2017). According to a study by the United Nations, OA affects approximately 7% of the global population, with more than 500 million people suffering from this condition (Hunter et al., 2020). Unfortunately, advanced surgical interventions, such as total joint arthroplasty, currently represent the most effective treatments for OA (Schwartz et al., 2020). Consequently, comprehending the etiology of this challenging disease is of utmost importance.

Psychiatric disorders, which include depression, bipolar disorder (Mullins et al., 2021), and schizophrenia (Goldfarb et al., 2022; Liu et al., 2021), constitute one of the leading causes of death worldwide (Mullins et al., 2021). Studies have found a noteworthy correlation between psychiatric disorders and osteoarthritis (OA), with these two conditions (Burant et al., 2023) often co‐occurring. Importantly, patients with psychiatric disorders undergoing total knee arthroplasty for OA experience more extended hospital stays and lower scores on the Knee Society Score (Stoica et al., 2022). The pain and fatigue associated with OA can also elevate the risk of depression, and assessing their response to nighttime pain and daily activities can aid in identifying those at risk (Hawker et al., 2011; Sugai et al., 2018). While bipolar disorder has been associated with endocrine and cardiovascular diseases such as diabetes (Kessing et al., 2004), dyslipidemia, and hypertension (Osborn et al., 2008; Rødevand et al., 2021), its prevalence in OA patients remains uncertain.

Psychiatric disorders can significantly impact the course of the disease, resulting in functional impairment and profoundly affecting the daily lives of individuals with osteoarthritis. They may also influence pain tolerance, potentially affecting the disease's diagnosis and treatment. Investigating whether psychiatric disorders serve as risk factors for OA can provide valuable insights into the etiological mechanisms of the disease and contribute to the development of novel management strategies.

Mendelian randomization (MR) is a statistical method based on genetic variation and is employed to explore causal relationships between exposure factors and outcome variables. This approach utilizes genetic variation in non‐experimental data to estimate the causal links between exposure factors and outcomes (Emdin et al., 2017). Unlike observational studies, MR employs genetic variation as an instrumental variable to mitigate drawbacks such as residual confounding and challenges in measuring exposure levels accurately. MR analysis is akin to a randomized controlled trial (RCT), considered the “gold standard” of medical evidence. Additionally, MR can balance known and unknown confounding factors in subgroups and mitigate inherent differences between exposure groups (Hemani et al., 2018).

Therefore, this project adopts a bidirectional approach for MR analysis to investigate the causal links between three distinct characteristic psychiatric disorders and the occurrence of OA.

## 2 METHODS

### 2.1 Study design

**FIGURE 1:**
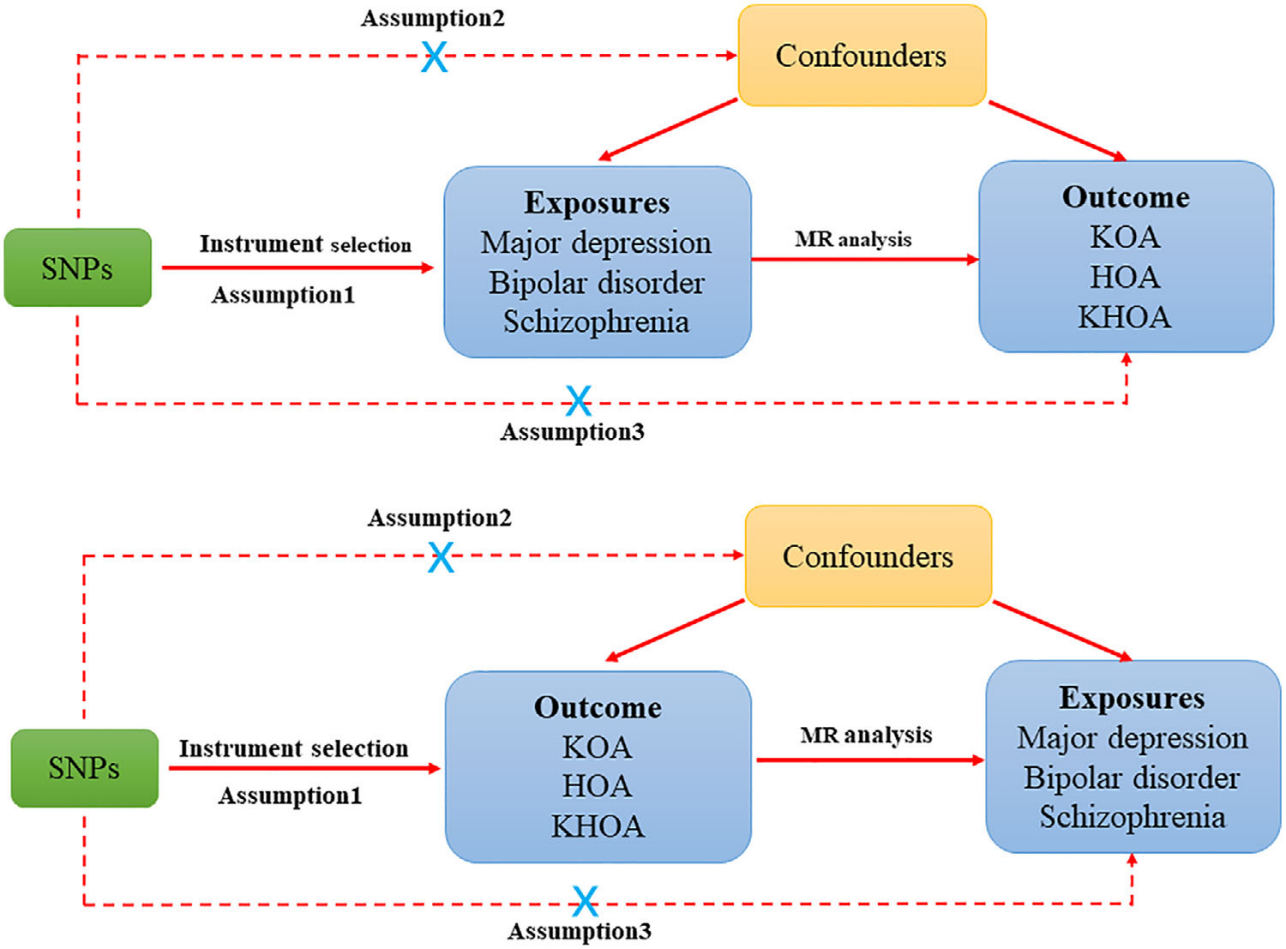
Schematic diagram of an MR causality study. Genetic variants are associated with KOA, HOA, and KHOA risk through psychiatric disorders only. Similar assumptions apply to the reverse MR analysis. MR, Mendelian randomization; KOA, knee osteoarthritis; HOA, hip osteoarthritis; KHOA, osteoarthritis of knee or hip; SNPs, single nucleotide polymorphisms.

Our study employed an MR design to investigate the causal relationship between psychiatric disorders and OA. Instrumental variables (IVs) were used in the MR analysis, with genetic variants, specifically single nucleotide polymorphisms (SNPs), selected as IVs. For a genetic variant to be considered suitable as an IV, it should be strongly associated with the exposure of interest, exhibit no association with potential confounding factors, or share common causes with the outcome variable (Didelez & Sheehan, 2007; Sanderson et al., 2022). Moreover, the genetic variant must influence the outcome solely through the exposure of interest. Figure 1 depicts the study design.

### 2.2 Data source

For this MR study, we utilized aggregated data from genome‐wide association studies (GWAS). The OA dataset was obtained from the UK Biobank and Arthritis Research UK Osteoarthritis Genetics (arcOGEN) and comprised 403,124 knee osteoarthritis (KOA) cases (24,955 cases and 378,169 controls), 393,873 hip osteoarthritis (HOA) cases (15,704 cases and 378,169 controls), and 417,596 osteoarthritis cases of the knee or hip (KHOA) (39,427 cases and 378,169 controls) (Tachmazidou et al., 2019). Data on bipolar disorder were collected from 57 cohorts comprising 41,917 cases of bipolar disorder and 371,549 controls of European ancestry from Europe, North America, and Australia (Mullins et al., 2021). GWAS aggregate statistics for major depression were extracted from the Psychiatric Genomics Consortium (PGC), including 500,199 samples with 170,756 cases and 329,443 controls (Howard et al., 2019). Similarly, summary statistics for GWAS on schizophrenia were obtained from the PGC, involving 320,404 samples (76,755 cases and 243,649 controls) (Pantelis et al., 2014). Further information about the data sources is presented in Table 1.

**TABLE 1:** Characteristics of the dataset used in this study.

Exposures	Outcomes	No. SNPs	Consortium	Sample size	Population
Major depression	HOA	40	PGC	500,199	European
	KOA	37			
	KHOA	37			
Bipolar disorder	HOA	35	PGC	413,466	European
	KOA	34			
	KHOA	34			
Schizophrenia	HOA	92	PGC	320,404	European
	KOA	91			
	KHOA	92			
HOA	Major depression	18	UK Biobank	393,873	European
	Bipolar disorder	21			
	Schizophrenia	15			
KOA	Major depression	5	UK Biobank	403,124	European
	Bipolar disorder	5			
	Schizophrenia	5			
KHOA	Major depression	21	UK Biobank	417,596	European
	Bipolar disorder	21			
	Schizophrenia	16			

Abbreviations: HOA, hip osteoarthritis; KHOA, osteoarthritis of knee or hip; KOA, knee osteoarthritis; PGC, Psychiatric Genomics Consortium; SNP, single‐nucleotide polymorphism .

All datasets used were publicly accessible, and ethical approval was obtained from the relevant institutions. Moreover, we ensured no overlap between the exposure and outcome samples to avoid any potential bias in the findings.

### 2.3 Selection of instrumental variables

Our study chose SNPs with genome‐wide significance (*p* < 5 × 10^−8^) as instrumental variables. A clumping algorithm was applied with an intercept value of *r* ^2^ = 0.001 to select all independently inherited instrumental SNPs. Additionally, we screened the underlying secondary phenotypes of each SNP that served as IVs using PhenoScanner. To ensure robustness, we re‐assessed the two‐sample MR analysis after excluding SNPs that might influence confounders. The following factors we considered as potential confounders in OA: body mass index (BMI), obesity, smoking, and bone mineral density (Jarecki et al., 2021; Pye et al., 2010; Ward & Dasgupta, 2020). All the mental diseases, history of mental illness, and sleep disturbances were excluded as confounders in psychiatric disorders (Haddock et al., 2016). SNPs in linkage disequilibrium (*r* ^2^ > 0.001) were excluded, and we harmonized the exposure and outcome variables for the effect allele in IVs across diverse databases. Furthermore, we performed the Mendelian randomization pleiotropy residual and outlier (MR‐PRESSO) test to recognize and eliminate pleiotropic variants. The intensity of each instrument was determined by investigating the F‐statistic, which was computed through the equation:

$$F=\frac{R^{2}\left(n-1-k\right)}{\left(1-R^{2}\right)k}$$

The F‐statistic was calculated from the sample size (*n*), the number of IVs (*k*), as well as the variance explained by IVs (*R* ^2^) (Pierce et al., 2011). We excluded instruments with an *F* < 10, which suggests weak mechanisms. We repeated the same procedures to investigate the reverse causal direction, obtaining IVs related to psychiatric disorders as outcomes while OA as exposures.

### 2.4 Mendelian randomization analysis

In our study, we employed five distinct MR analyses: the Mendelian randomization‐Egger regression (MR‐Egger) method, the weighted median method, the inverse variance weighted (IVW) method, the simple mode method, and the weighted mode method. Among these, the IVW method is considered the most robust approach for MR analysis (Burgess & Thompson, 2011). To account for SNPs as instrumental variables in MR analysis and determine their associations with other exposures, we used the IVW and MR‐Egger methods. Bonferroni's correction was performed to adjust the *p*‐values (*p* < 0.0056 = 0.05/9 for univariate MR analyses and *p* < 0.0033 = 0.05/15 for multivariate MR analyses). Heterogeneity was assessed using the Cochran Q statistic, with Cochran's Q‐derived *p <* 0.05 indicating evidence of heterogeneity (Bowden et al., 2017). Genetic variants may impact the results due to potential pleiotropy effects, leading to bias. To address this bias arising from horizontal pleiotropy, we utilized the MR‐Egger approach, which provides an estimation of the magnitude of directional pleiotropy.

To test the robustness of the MR analysis, we conducted a “leave‐one‐out” sensitivity analysis. This involved removing non‐specific SNPs from the analysis and considering the causal evidence between exposure and outcome to be more substantial if the association between other instrumental variables and the outcome remained statistically significant. We visually assessed the robustness of the findings by iteratively removing SNPs one by one, reanalyzing the results, and presenting them in the form of forest plots. This allowed us to observe the impact of individual SNPs on the overall MR analysis and confirm the stability of our results.

We were considering additional confounding factors such as BMI, smoking, and bone mineral density, which may increase the risk of OA. We conducted multivariate Mendelian randomization (MVMR) analyses including BMI and smoking as exposures to examine the potential causal links between psychiatric disorders and OA. TwoSampleMR, MR‐PRESSO, and MVMR packages of R (version 4.2.2) were used to perform all analyses (Zheng et al., 2017).

## 3 RESULTS

### 3.1 The causal effect of psychiatric disorders on OA

**FIGURE 2:**
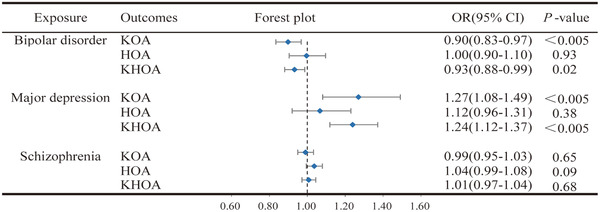
Mendelian randomization analysis explores the causal link between psychiatric disorders as an exposure factor and osteoarthritis as an outcome.

We conducted univariable and multivariable MR analyses on bipolar disorder and OA. After removing the linkage disequilibrium and 14 SNPs associated with confounding factors, we identified the exposure (bipolar disorder) and outcome (KOA, HOA, or KHOA) SNPs and removed the palindromic sequences. Finally, the obtained valid IVs are presented in Tables S1–S3. Then, a total of 34 SNPs related to bipolar disorder were determined, revealing a conserved causal link between bipolar disorder and KOA (OR = 0.90, 95% CI = 0.83 to 0.97, *p* = 0.0048, Figure 2 and Figure S1A). This means that the risk of KOA decreases by 0.90 per unit increase in log‐odds of bipolar disorder. Similarly, potential evidence was observed for a conserved causal link between bipolar disorder and KHOA (OR = 0.93, 95% CI = 0.88 to 0.99, *p* = 0.02, Figure 2 and Figure S1C). However, evidence of the positive finding after being adjusted by the Bonferroni correction was only suggestive. Unfortunately, when conducting MVMR analysis, the casual estimates were no longer sustained between bipolar disorder and KOA.

Regarding the relationships between major depression and KOA, the IVW (OR = 1.27; 95% CI = 1.08 to 1.49; *p* = 0.0036, Figure 2) and weighted median methods (OR = 1.32; 95% CI = 1.11 to 1.56; *p* = 0.001, Figure S1D) showed a positive causal influence of major depression on KOA. The study results concerning the causal influence of major depression and KHOA, listed in Figure 2, demonstrated that major depression had a positive causal relationship with KHOA based on the IVW (OR = 1.24; 95% CI = 1.12 to 1.37; *p* = 3.62E‐05) and weighted median analysis (OR = 1.22; 95% CI = 1.07 to 1.40; *p* = 0.004, Figure S1F). These results demonstrate that major depression is a risk factor for KOA and KHOA and that patients with major depression increase the probability of KOA or KHOA. No remarkable causal relationship between major depression and HOA (*p* = 0.38, Figure S1E). In the MVMR analyses, major depression was found to be causally associated with a significantly high risk of KHOA after adjusting BMI (OR = 1.44; 95% CI = 1.09 to 1.81; *p* = 8.56E‐04, Figure 4) or smoking (OR = 1.46; 95% CI = 1.19 to 1.65; *p* = 0.0032, Figure 4), which was similar to the two sample MR result. However, there was no causal association between major depression and KOA in MVMR.

Additionally, we investigated the causal effect between schizophrenia and OA. Unfortunately, according to the IVW analysis, it was not evidenced that there was a causal link between schizophrenia and KOA (*p =* 0.65), HOA (*p =* 0.09), or KHOA (*p =* 0.68) (Figure 2). Moreover, MVMR analyses demonstrated no causal effect between schizophrenia and KOA, HOA, or KHOA (Figure 4).

The sensitivity analysis showed no heterogeneity (*p >* 0.05) in the major depression‐HOA, major depression‐KHOA, schizophrenia‐HOA, KOA‐major depression, and KHOA‐bipolar disorder. There was heterogeneity among others. No horizontal pleiotropy (*p* > 0.05) was noted in the psychiatric disorders‐OA and OA‐psychiatric disorders subgroup (Table 2). To exclude the likelihood that any single instrument might be affecting the results of point estimates or statistical examinations strongly, a “leave‐one‐out” analysis was executed, which demonstrated that the absence of any single instrument acting as an outlier affected the causal influence estimates, suggesting no fundamental effect of the removal of any SNPs on the results (Figure S2). The forest plot for causal effects of psychiatric disorders on OA is shown in Figure S3. Evidence of weak IVs bias was absent in every sample (*F* statistic > 10, Tables S1–S9).

**TABLE 2:** Heterogeneity and horizontal pleiotropy of the associations between psychiatric disorders and OA (KOA, HOA, and KHOA).

		Heterogeneity test (IVW)		Heterogeneity test (MR Egger)		MR Egger intercept test		
Exposures	Outcomes	Q‐value	*p*	Q‐value	*p*	I	SE	*p*
Bipolar disorder	KOA	62.78	.00	62.35	.00	0.00	0.01	.64
	HOA	69.83	<.01	69.79	<.01	0.00	0.02	.90
	KHOA	55.13	.01	55.09	.01	0.00	0.01	.89
Major depression	KOA	70.00	.00	62.79	.00	0.30	0.02	.06
	HOA	38.30	.41	35.01	.51	‐0.03	0.01	.07
	KHOA	43.63	.18	43.60	.15	0.00	0.00	.88
schizophrenia	KOA	167.52	<.01	165.28	<.01	0.00	0.00	.27
	HOA	90.74	.48	90.42	.47	0.00	0.00	.58
	KHOA	165.99	<.01	163.66	<.01	0.00	0.00	.26
KOA	Bipolar disorder	10.89	.03	8.34	.04	0.03	0.04	.41
	Major depression	0.96	.92	0.77	.86	0.00	0.00	.70
	schizophrenia	14.74	.00	14.42	.00	0.01	0.04	.81
HOA	Bipolar disorder	32.68	.03	30.74	.04	‐0.01	0.01	.29
	Major depression	32.53	.01	31.90	.01	0.00	0.00	.56
	schizophrenia	53.70	<.01	53.63	<.01	0.00	0.01	.88
KHOA	Bipolar disorder	27.92	.11	27.86	.09	0.00	0.02	.83
	Major depression	40.12	.00	40.02	.00	0.00	0.00	.83
	schizophrenia	35.73	.00	34.80	.00	0.01	0.02	.55

Abbreviations: IVW, inverse variance weighted; KHOA, osteoarthritis of knee or hip; KOA, knee osteoarthritis; HOA, hip osteoarthritis; MR, Mendelian randomization.

### 3.2 The causal effect of OA on psychiatric disorders

In addition to investigating the causal effect of psychiatric disorders on OA, we conducted a reverse MR analysis to explore the causal influence of KOA, HOA, and KHOA on psychiatric disorders.

Using a stringent criterion of *p* < 5 × 10^−8^, we selected instrumental variables (IVs) for the MR analysis. Evidence of weak IVs was not found, further supporting the reliability of the IVs selected. The number of relevant SNPs used in the MR analysis for each type of OA as an exposure factor is presented in Tables S10–S18 and Figure S4.

**FIGURE 3:**
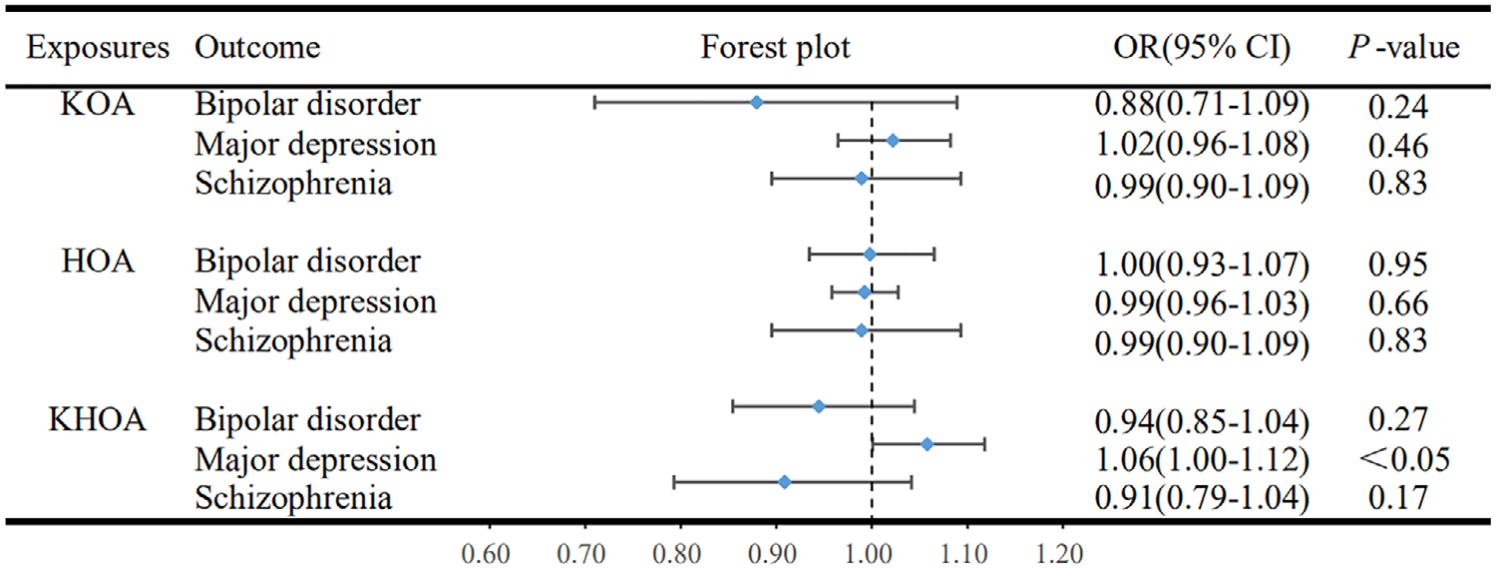
Inverse Mendelian randomization analyses explore the causal impact of knee osteoarthritis, hip osteoarthritis, and osteoarthritis of knee or hip as exposure factors on bipolar disorder, major depression, or schizophrenia.

The findings revealed that only KHOA demonstrated a suggestive causal relationship with the incidence of major depression. KHOA was found to be a potential risk factor for major depression, increasing its probability of occurrence (OR = 1.06; 95% CI = 1.00 to 1.12; *p* = 0.0469, Figure 3). However, no evidence of a causal relationship was observed between KHOA and bipolar disorder (*p* = 0.27) or schizophrenia (*p* = 0.17). Moreover, no statistical effect of KOA on the incidence of bipolar disorder (*p* = 0.24), major depression (*p* = 0.46), or schizophrenia (*p* = 0.83) was found. Similarly, there was no evidence of a causal effect of HOA on bipolar disorder (*p* = 0.95), major depression (*p* = 0.66), or schizophrenia (*p* = 0.83), indicating that HOA does not significantly impact the probability of developing psychiatric disorders (Figure 3).

**FIGURE 4:**
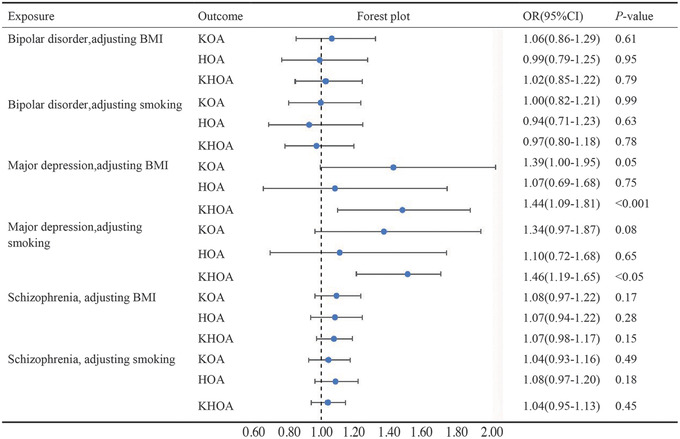
Multivariate Mendelian randomization analyses explore the causal impact of knee osteoarthritis, hip osteoarthritis, and osteoarthritis of knee or hip as exposure factors on bipolar disorder, major depression, or schizophrenia after adjusting body mass index or smoking.

Furthermore, the reverse MR sensitivity analysis revealed no horizontal pleiotropy (*p >* 0.05). There was no heterogeneity between the relationship of KOA with major depression (*p* > 0.05) and the relationship of KHOA with bipolar depression (*p* > 0.05). However, heterogeneity was observed among other relationships (*p* < 0.05, Table 2). Additionally, the “leave‐one‐out” analysis indicated that no individual SNP significantly influenced the overall estimate (Figure S5), further supporting the robustness of our results. The forest plot illustrating the causal effects of OA (KOA, HOA, or KHOA) on psychiatric disorders is shown in Figure S6.

## 4 DISCUSSION

This study represents the first attempt to utilize GWAS datasets to assess the causal impact of bipolar disorder, major depression, and schizophrenia on KOA, HOA, or KHOA using a bidirectional MR analysis. The results of our study demonstrate that major depression acts as a risk factor for KHOA. In exploring the causal links between OA and psychiatric disorders, we only identified KHOA as a potential risk factor for major depression.

Previous MR research suggested a causal relationship between bipolar disorder and rheumatoid arthritis (RA), with bipolar disorder acting as a protective factor for RA (Xiang et al., 2023). However, no causal association between bipolar disorder and OA has been reported, making our study a pioneering effort to bridge this knowledge gap. The results of our MR analysis provide compelling evidence for a causal genetic link between bipolar disorder and KOA risk. Several related investigations have highlighted the significance of Ca^2+^ in neurotransmitter release within the brain in bipolar disorder. The dysfunction of cellular Ca^2+^ channels has been identified as a major contributor to pathological clinical behavior (Andrade et al., 2019; Harrison et al., 2018) in bipolar disorder. Similarly, the disruption of Ca^2+^ signaling has been recognized as a key mechanism in the pathogenesis of KOA. Excessive Ca^2+^ inward flow in chondrocytes, elevated resting Ca^2+^ concentration, and increased sensitivity of chondrocytes to external mechanical forces lead to a fragile chondrocyte skeleton, making it more susceptible to mechanical trauma and exacerbating the development of KOA (Lee et al., 2014). Moreover, excessive Ca^2+^ inward flow further promotes an inflammatory response in chondrocytes, contributing to the worsening KOA severity (Lee et al., 2014, 2021; Zhong et al., 2021). Therefore, the dysregulated Ca^2+^ regulatory balance in patients with bipolar disorder may explain the lower incidence of KOA observed in these individuals. Additionally, a well‐established dopamine hypothesis has been associated with bipolar disorder for nearly four decades. Excessive dopamine levels have been observed in bipolar disorder patients, potentially linked to impaired dopamine transporter protein function, and elevated D2/3 receptor availability in the striatum (Ashok et al., 2017; Milienne‐Petiot et al., 2017). Interestingly, using hyaluronic acid combined with dopamine cavity injections has effectively increased cartilage lubrication, reduced cartilage friction, and halted the progression of KOA (Ren et al., 2022). As a neurotransmitter, dopamine plays a significant role in regulating exercise, cardiovascular activity, and gastrointestinal function and is a vital molecule linking neural activity and immune response (Basu & Dasgupta, 2000; Sarkar et al., 2010). Animal models with dopamine receptor D2 (DRD2) knockout showed significant inflammatory reactions, while dopamine intervention in macrophages resulted in significant inhibition of the activation of DLRP3 inflammatory vesicles (Yan et al., 2015). Therefore, the excessive release of dopamine may potentially have a protective effect on KOA and KHOA patients with bipolar disorder through specific mechanisms, potentially inhibiting inflammatory responses and disrupting the inflammation‐mediated cartilage damage and apoptosis cascade.

Major depression, as a psychiatric disorder, has been associated with inflammatory activity processes in both the peripheral and central nervous systems (Lee et al., 2013). Coincidentally, both KOA and KHOA are characterized by elevated levels of inflammation, leading to various pathological changes (Bonnet & Walsh, 2005). This suggests that there may be shared pathological features or pathogenetic links between major depression and osteoarthritis, and our study confirms this, with major depression demonstrating significant significance as a risk factor for KHOA both in our univariate and multivariate MR analyses. Moreover, previous research has indicated significant genome‐wide genetic correlations and a common etiology (Barowsky et al., 2021) between major depression and osteoarthritis. Our study's advantage lies in further classifying osteoarthritis into KOA and HOA based on the site of onset, which allows for more comprehensive and systematic etiological diagnosis and clinical treatment. In recent years, emerging studies have revealed that pathological behaviors of major depression are associated with abnormal activity in brain regions such as the amygdala and frontal cortex. The amygdala, a processing center that regulates mood, fear, emotional states, and pain perception, may exhibit abnormalities in function, leading to various subtypes of depression (Brown et al., 2020; Lindquist et al., 2012). Similarly, the expression of pain intensity in osteoarthritis is associated with blood supply to the anterior cingulate cortex and amygdala regions of the brain (Cottam et al., 2016), suggesting that the same brain regions regulate pain perception in both major depression and OA and that the pathogenesis of the two conditions may be interrelated.

On the other hand, in the reverse MR analysis, we found suggestive evidence of a positive causal link between KHOA and major depression. KHOA may act as a potential risk factor for patients with major depression. Mounting evidence suggests that chronic inflammatory activity is strongly associated with an increased risk of developing major depression through mechanisms such as increased peripheral immune action, continuous production of proinflammatory regulators interleukin‐1 (IL‐1β), tumor necrosis factor (TNF), and interleukin‐6 (IL‐6) by microglia in the brain, and reduced production of anti‐inflammatory factors (Dilger & Johnson, 2008). Recent observational studies and meta‐analyses have also confirmed the link between major depression and inflammation, with inflammation being both a cause and a consequence of major depression (Chae et al., 2023; Ng & Ellman, 2021). Notably, inflammation is a crucial pathological change in KHOA. Proinflammatory factors (PIC), including interleukins (IL), matrix metalloproteinases (MMP), and tumor necrosis factors (TNF), have been identified as essential mediators of the inflammatory response leading to the development and progression of KHOA (Kapoor et al., 2011; Mabey & Honsawek, 2015). Consequently, we speculate that KHOA may act as a risk factor for major depression through its association with the inflammatory response. In patients with KHOA, increased inflammatory activity disrupts the balance between cellular inflammatory and anti‐inflammatory factors, influencing the release of neurotransmitters in specific brain regions and thereby promoting the development of major depression.

Despite the valuable insights gained from this study, there are some limitations. First, our sample data are derived solely from European individuals, which may not represent more diverse populations beyond non‐European ethnicities. Additionally, selecting only three types of psychiatric disorders for exploration may not capture the full range of possible relationships with OA. The two‐sample MR approach in this work may introduce sample overlap, potentially increasing the likelihood of weak IV bias. Furthermore, our sensitivity analysis revealed different degrees of heterogeneity, indicating that our findings may be impacted by pleiotropy.

## 5 CONCLUSION

Our MR analysis, using GWAS data on psychiatric disorders (bipolar disorder, major depression, and schizophrenia) and OA subtypes (KOA, HOA, and KHOA), has yielded important insights into the causal relationships between these conditions. We found that major depression has a positive causal association with KHOA. Moreover, our reverse MR analysis suggests that KHOA may be a potential risk factor for major depression. However, we did not find any genetic evidence to support KOA, HOA, or KHOA as causative factors for bipolar disorder or schizophrenia. These findings provide valuable evidence for further etiological and epidemiological investigations of the association between psychiatric disorders and OA.

Identifying causal links between these conditions can potentially inform the development of targeted interventions and improve clinical management for individuals affected by psychiatric disorders and osteoarthritis. Further research is warranted to validate and expand upon these findings and to explore the underlying molecular mechanisms connecting these conditions.

## AUTHOR CONTRIBUTIONS

**Jinzhi Meng**: Conceptualization; data curation; formal analysis; writing—original draft. **Youran Cai**: Data curation; formal analysis; writing—original draft. **Jun Yao**: Writing—review and editing; funding acquisition; supervision; methodology. **Haiwei Yan**: Writing—review and editing; methodology; data curation; supervision.

## CONFLICT OF INTEREST STATEMENT

The authors declare no conflicts of interest.

### PEER REVIEW

The peer review history for this article is available at https://publons.com/publon/10.1002/brb3.3429

## Supporting information

Figure S1: Scatter plot regarding the causal effect of psychiatric disorders on OA. Scatter plot of bipolar disorder on KOA (A), HOA (B) and KHOA (C); major depression on KOA (D), HOA (E) and KHOA (F); schizophrenia on KOA (G), HOA (H) and KHOA (I). KOA, Knee OA; HOA, Hip OA; KHOA, Osteoarthritis of knee or hip.
Figure S2: Leave‐one‐out analysis for the causal effect of psychiatric disorders on OA. Leave‐one‐out analysis of bipolar disorder on KOA (A), HOA (B) and KHOA (C); major depression on KOA (D), HOA (E) and KHOA (F); schizophrenia on KOA (G), HOA (H) and KHOA (I). KOA, Knee OA; HOA, Hip OA; KHOA, Osteoarthritis of knee or hip.
Figure S3: Forest plot for the causal effects of psychiatric disorders on OA. (A) Forest plot for the causal effects of bipolar disorder on KOA (A), HOA (B) and KHOA (C); major depression on KOA (D), HOA (E) and KHOA (F); schizophrenia on KOA (G), HOA (H) and KHOA (I). KOA, Knee OA; HOA, Hip OA; KHOA, Osteoarthritis of knee or hip.
Figure S4: Scatter plot regarding the causal effect of OA on psychiatric disorders in the inverse MR Analysis. Scatter plots of causal effects of KOA on bipolar disorder (A), major depression (B), and schizophrenia (C); HOA on bipolar disorder (D), major depression (E), and schizophrenia (F); KHOA on bipolar disorder (G), major depression (H), and schizophrenia (I). KOA, Knee OA; HOA, Hip OA; KHOA, Osteoarthritis of knee or hip.
Figure S5: Leave‐one‐out analysis for the causal effect of OA on psychiatric disorders in the inverse MR Analysis. Leave‐one‐out analysis for the causal effect of KOA on bipolar disorder (A), major depression (B), and schizophrenia (C); HOA on bipolar disorder (D), major depression (E), and schizophrenia (F); KHOA on bipolar disorder (G), major depression (H), and schizophrenia (I). KOA, Knee OA; HOA, Hip OA; KHOA, Osteoarthritis of knee or hip.
Figure S6: Forest plot for the causal effects of OA on psychiatric disorders in the inverse MR Analysis. Forest plot for the overall causal effects of KOA on bipolar disorder (A), major depression (B), and schizophrenia (C); HOA on bipolar disorder (D), major depression (E), and schizophrenia (F); KHOA on bipolar disorder (G), major depression (H), and schizophrenia (I). KOA, Knee OA; HOA, Hip OA; KHOA, Osteoarthritis of knee or hip.

Table S1. 34 valid IVs used for MR analysis of bipolar disorder on KOA
Table S2. 35 valid IVs used for MR analysis of bipolar disorder on HOA
Table S3. 34 valid IVs used for MR analysis of bipolar disorder on KHOA
Table S4. 5 valid IVs used for MR analysis of KOA on bipolar disorder
Table S5. 21valid IVs used for MR analysis of HOA on bipolar disorder
Table S6. 21 valid IVs used for MR analysis of KHOA on bipolar disorder
Table S7. 37 valid IVs used for MR analysis of major depression on KOA
Table S8. 38 valid IVs used for MR analysis of major depression on HOA
Table S9. 37 valid IVs used for MR analysis of major depression on KHOA
Table S10. 5 valid IVs used for MR analysis of KOA on major depression
Table S11. 18 valid IVs used for MR analysis of HOA on major depression
Table S12. 21 valid IVs used for MR analysis of KHOA on major depression
Table S13. 91 valid IVs used for MR analysis of schizophrenia on KOA
Table S14. 92 valid IVs used for MR analysis of schizophrenia on HOA
Table S15. 92 valid IVs used for MR analysis of schizophrenia on KHOA
Table S16. 5 valid IVs used for MR analysis of KOA on schizophrenia
Table S17. 15 valid IVs used for MR analysis of HOA on schizophrenia
Table S18. 16 valid IVs used for MR analysis of KHOA on schizophrenia.

## Data Availability

The summary data of OA GWAS can be downloaded from the Integrative Epidemiology Unit (IEU) GWAS database (https://gwas.mrcieu.ac.uk), and the psychiatric disorder GWAS can be obtained from the Psychiatric Genomics Consortium (https://pgc.unc.edu/for‐researchers).

## References

[brb33429-bib-0029] Andrade, A. , Brennecke, A. , Mallat, S. , Brown, J. , Gomez‐Rivadeneira, J. , Czepiel, N. , & Londrigan, L. (2019). Genetic associations between voltage‐gated calcium channels and psychiatric disorders. International Journal of Molecular Sciences, 20(14), 3537. 10.3390/ijms20143537 31331039 PMC6679227

[brb33429-bib-0022] Ashok, A. H. , Marques, T. R. , Jauhar, S. , Nour, M. M. , Goodwin, G. M. , Young, A. H. , & Howes, O. D. (2017). The dopamine hypothesis of bipolar affective disorder: The state of the art and implications for treatment. Molecular Psychiatry, 22(5), 666–679. 10.1038/mp.2017.16 28289283 PMC5401767

[brb33429-bib-0039] Barowsky, S. , Jung, J.‐Y. , Nesbit, N. , Silberstein, M. , Fava, M. , Loggia, M. L. , Smoller, J. W. , & Lee, P. H. (2021). Cross‐disorder genomics data analysis elucidates a shared genetic basis between major depression and osteoarthritis pain. Frontiers in Genetics, 12, 687687. 10.3389/fgene.2021.687687 34603368 PMC8481820

[brb33429-bib-0001] Basu, S. , & Dasgupta, P. S. (2000). Dopamine, a neurotransmitter, influences the immune system. Journal of Neuroimmunology, 102(2), 113–124. 10.1016/S0165-5728(99)00176-9 10636479

[brb33429-bib-0003] Bonnet, C. S. , & Walsh, D. A. (2005). Osteoarthritis, angiogenesis and inflammation. Rheumatology, 44(1), 7–16. 10.1093/rheumatology/keh344 15292527

[brb33429-bib-0020] Bowden, J. , Del Greco, M. F. , Minelli, C. , Davey Smith, G. , Sheehan, N. , & Thompson, J. (2017). A framework for the investigation of pleiotropy in two‐sample summary data Mendelian randomization. Statistics in Medicine, 36(11), 1783–1802. 10.1002/sim.7221 28114746 PMC5434863

[brb33429-bib-0033] Brown, S. S. G. , Rutland, J. W. , Verma, G. , Feldman, R. E. , Schneider, M. , Delman, B. N. , Murrough, J. M. , & Balchandani, P. (2020). Ultra‐high‐resolution imaging of amygdala subnuclei structural connectivity in major depressive disorder. Biological Psychiatry Cognitive Neuroscience and Neuroimaging, 5(2), 184–193. 10.1016/j.bpsc.2019.07.010 31570286 PMC7010542

[brb33429-bib-0010] Burgess, S. , & Thompson, S. G. (2011). Avoiding bias from weak instruments in Mendelian randomization studies. International Journal of Epidemiology, 40(3), 755–764. 10.1093/ije/dyr036 21414999

[brb33429-bib-0049] Burant, C. J. , Graham, G. C. , Deimling, G. , Kresevic, D. , Kahana, E. , Wykle, M. , Kwoh, C. K. , & Ibrahim, S. A. (2023). The effects of osteoarthritis on depressive symptomatology among older U.S. military veterans. International Journal of Aging and Human Development, 96(3), 267–284. 35285279 10.1177/00914150221084952

[brb33429-bib-0046] Chae, W. R. , Baumert, J. , Nübel, J. , Brasanac, J. , Gold, S. M. , Hapke, U. , & Otte, C. (2023). Associations between individual depressive symptoms and immunometabolic characteristics in major depression. European Neuropsychopharmacology, 71, 25–40. 10.1016/j.euroneuro.2023.03.007 36966710

[brb33429-bib-0017] Cottam, W. J. , Condon, L. , Alshuft, H. , Reckziegel, D. , & Auer, D. P. (2016). Associations of limbic‐affective brain activity and severity of ongoing chronic arthritis pain are explained by trait anxiety. NeuroImage: Clinical, 12, 269–276. 10.1016/j.nicl.2016.06.022 27504262 PMC4969259

[brb33429-bib-0004] Didelez, V. , & Sheehan, N. (2007). Mendelian randomization as an instrumental variable approach to causal inference. Statistical Methods in Medical Research, 16(4), 309–330. 10.1177/0962280206077743 17715159

[brb33429-bib-0006] Dilger, R. N. , & Johnson, R. W. (2008). Aging, microglial cell priming, and the discordant central inflammatory response to signals from the peripheral immune system. Journal of Leukocyte Biology, 84(4), 932–939. 10.1189/jlb.0208108 18495785 PMC2538600

[brb33429-bib-0019] Emdin, C. A. , Khera, A. V. , & Kathiresan, S. (2017). Mendelian randomization. JAMA, 318(19), 1925–1926. 10.1001/jama.2017.17219 29164242

[brb33429-bib-0041] Goldfarb, M. , De Hert, M. , Detraux, J. , Di Palo, K. , Munir, H. , Music, S. , Piña, I. , & Ringen, P. A. (2022). Severe mental illness and cardiovascular disease: JACC state‐of‐the‐art review. Journal of the American College of Cardiology, 80(9), 918–933. 10.1016/j.jacc.2022.06.017 36007991

[brb33429-bib-0016] Haddock, G. , Davies, L. , Evans, E. , Emsley, R. , Gooding, P. , Heaney, L. , Jones, S. , Kelly, J. , Munro, A. , Peters, S. , Pratt, D. , Tarrier, N. , Windfuhr, K. , & Awenat, Y. (2016). Investigating the feasibility and acceptability of a cognitive behavioural suicide prevention therapy for people in acute psychiatric wards (the ‘INSITE’ trial): Study protocol for a randomised controlled trial. Trials, 17, 79. 10.1186/s13063-016-1192-9 26869076 PMC4751630

[brb33429-bib-0026] Harrison, P. J. , Geddes, J. R. , & Tunbridge, E. M. (2018). The emerging neurobiology of bipolar disorder. Trends in Neurosciences, 41(1), 18–30. 10.1016/j.tins.2017.10.006 29169634 PMC5755726

[brb33429-bib-0047] Hawker, G. A. , Gignac, M. A. , Badley, E. , Davis, A. M. , French, M. R. , Li, Y. , Perruccio, A. V. , Power, J. D. , Sale, J. , & Lou, W. (2011). A longitudinal study to explain the pain‐depression link in older adults with osteoarthritis. Arthritis Care & Research, 63(10), 1382–1390. 20662042 10.1002/acr.20298

[brb33429-bib-0025] Hemani, G. , Zheng, J. , Elsworth, B. , Wade, K. H. , Haberland, V. , Baird, D. , Laurin, C. , Burgess, S. , Bowden, J. , Langdon, R. , Tan, V. Y. , Yarmolinsky, J. , Shihab, H. A. , Timpson, N. J. , Evans, D. M. , Relton, C. , Martin, R. M. , Smith, G. D. , Gaunt, T. R. , … Haycock, P. C. (2018). The MR‐Base platform supports systematic causal inference across the human phenome. Elife, 7, e34408. 10.7554/eLife.34408 29846171 PMC5976434

[brb33429-bib-0028] Howard, D. M. , Adams, M. J. , Clarke, T.‐K. , Hafferty, J. D. , Gibson, J. , Shirali, M. , Coleman, J. R. I. , Hagenaars, S. P. , Ward, J. , Wigmore, E. M. , Alloza, C. , Shen, X. , Barbu, M. C. , Xu, E. Y. , Whalley, H. C. , Marioni, R. E. , Porteous, D. J. , Davies, G. , Deary, I. J. , … Mcintosh, A. M. (2019). Genome‐wide meta‐analysis of depression identifies 102 independent variants and highlights the importance of the prefrontal brain regions. Nature Neuroscience, 22(3), 343–352. 10.1038/s41593-018-0326-7 30718901 PMC6522363

[brb33429-bib-0030] Hunter, D. J. , March, L. , & Chew, M. (2020). Osteoarthritis in 2020 and beyond: A Lancet Commission. The Lancet, 396(10264), 1711–1712. 10.1016/S0140-6736(20)32230-3 33159851

[brb33429-bib-0037] Jarecki, J. , Małecka‐Massalska, T. , Polkowska, I. , Potoczniak, B. , Kosior‐Jarecka, E. , Szerb, I. , Tomaszewska, E. , Gutbier, M. , Dobrzyński, M. , & Blicharski, T. (2021). Level of adiponectin, leptin and selected matrix metalloproteinases in female overweight patients with primary gonarthrosis. Journal of Clinical Medicine, 10(6), 1263. 10.3390/jcm10061263 33803785 PMC8003316

[brb33429-bib-0011] Kapoor, M. , Martel‐Pelletier, J. , Lajeunesse, D. , Pelletier, J.‐P. , & Fahmi, H. (2011). Role of proinflammatory cytokines in the pathophysiology of osteoarthritis. Nature Reviews Rheumatology, 7(1), 33–42. 10.1038/nrrheum.2010.196 21119608

[brb33429-bib-0002] Kessing, L. V. , Nilsson, F. M. , Siersma, V. , & Kragh Andersen, P. (2004). Increased risk of developing diabetes in depressive and bipolar disorders? Journal of Psychiatric Research, 38(4), 395–402. 10.1016/j.jpsychires.2003.12.001 15203291

[brb33429-bib-0048] Lee, S. H. , Ripke, S. , Neale, B. M. , Faraone, S. V. , Purcell, S. M. , Perlis, R. H. , Mowry, B. J. , Thapar, A. , Goddard, M. E. , Witte, J. S. , Absher, D. , Agartz, I. , Akil, H. , Amin, F. , Andreassen, O. A. , Anjorin, A. , Anney, R. , Anttila, V. , Arking, D. E. , … International Inflammatory Bowel Disease Genetics Consortium (IIBDGC) . (2013). Genetic relationship between five psychiatric disorders estimated from genome‐wide SNPs. Nature Genetics, 45(9), 984–994. 23933821 10.1038/ng.2711 PMC3800159

[brb33429-bib-0050] Lee, W. , Leddy, H. A. , Chen, Y. , Lee, S. H. , Zelenski, N. A. , McNulty, A. L. , Wu, J. , Beicker, K. N. , Coles, J. , Zauscher, S. , & Grandl, J. (2014). Synergy between Piezo1 and Piezo2 channels confers high‐strain mechanosensitivity to articular cartilage. Proceedings of the National Academy of Sciences of the United States of America, 111(47), E5114–E5122. 25385580 10.1073/pnas.1414298111 PMC4250098

[brb33429-bib-0052] Lee, W. , Nims, R. J. , Savadipour, A. , Zhang, Q. , Leddy, H. A. , Liu, F. , McNulty, A. L. , Chen, Y. , Guilak, F. , & Liedtke, W. B. (2021). Inflammatory signaling sensitizes Piezo1 mechanotransduction in articular chondrocytes as a pathogenic feed‐forward mechanism in osteoarthritis. Proceedings of the National Academy of Sciences of the United States of America, 118(13), e2001611118. 33758095 10.1073/pnas.2001611118 PMC8020656

[brb33429-bib-0018] Li, M. H. , Xiao, R. , Li, J. B. , & Zhu, Q. (2017). Regenerative approaches for cartilage repair in the treatment of osteoarthritis. Osteoarthritis and Cartilage, 25(10), 1577–1587. 10.1016/j.joca.2017.07.004 28705606

[brb33429-bib-0012] Lindquist, K. A. , Wager, T. D. , Kober, H. , Bliss‐Moreau, E. , & Barrett, L. F. (2012). The brain basis of emotion: A meta‐analytic review. The Behavioral and Brain Sciences, 35(3), 121–143. 10.1017/S0140525X11000446 22617651 PMC4329228

[brb33429-bib-0035] Liu, C. , Pan, W. , Li, L. , Li, B. , Ren, Y. , & Ma, X. (2021). Prevalence of depression, anxiety, and insomnia symptoms among patients with COVID‐19: A meta‐analysis of quality effects model. Journal of Psychosomatic Research, 147, 110516. 10.1016/j.jpsychores.2021.110516 34023580 PMC8129994

[brb33429-bib-0015] Mabey, T. , & Honsawek, S. (2015). Cytokines as biochemical markers for knee osteoarthritis. World Journal of Orthopedics, 6(1), 95–105. 10.5312/wjo.v6.i1.95 25621214 PMC4303794

[brb33429-bib-0023] Milienne‐Petiot, M. , Groenink, L. , Minassian, A. , & Young, J. W. (2017). Blockade of dopamine D_1_‐family receptors attenuates the mania‐like hyperactive, risk‐preferring, and high motivation behavioral profile of mice with low dopamine transporter levels. Journal of Psychopharmacology, 31(10), 1334–1346. 10.1177/0269881117731162 28950781 PMC10773978

[brb33429-bib-0034] Mullins, N. , Forstner, A. J. , O'connell, K. S. , Coombes, B. , Coleman, J. R. I. , Qiao, Z. , Als, T. D. , Bigdeli, T. B. , Børte, S. , Bryois, J. , Charney, A. W. , Drange, O. K. , Gandal, M. J. , Hagenaars, S. P. , Ikeda, M. , Kamitaki, N. , Kim, M. , Krebs, K. , Panagiotaropoulou, G. , … Andreassen, O. A. (2021). Genome‐wide association study of more than 40,000 bipolar disorder cases provides new insights into the underlying biology. Nature Genetics, 53(6), 817–829. 10.1038/s41588-021-00857-4 34002096 PMC8192451

[brb33429-bib-0040] Ng, T. H. , & Ellman, L. M. (2021). The longitudinal associations of inflammatory biomarkers and depression revisited: Systematic review, meta‐analysis, and meta‐regression. Molecular Psychiatry, 26(7), 3302–3314. 32807846 10.1038/s41380-020-00867-4 PMC7887136

[brb33429-bib-0005] Osborn, D. P. J , Wright, C. A. , Levy, G. , King, M. B. , Deo, R. , & Nazareth, I. (2008). Relative risk of diabetes, dyslipidaemia, hypertension and the metabolic syndrome in people with severe mental illnesses: Systematic review and metaanalysis. BMC Psychiatry, 8, 84. 10.1186/1471-244X-8-84 18817565 PMC2570660

[brb33429-bib-0051] Pantelis, C. , Papadimitriou, G. N. , Papiol, S. , Parkhomenko, E. , Pato, M. T. , Paunio, T. , Pejovic‐Milovancevic, M. , Perkins, D. O. , Pietiläinen, O. , Pimm, J. , & O'Donovan, M. C. (2014). Biological insights from 108 schizophrenia‐associated genetic loci. Nature, 511(7510), 421–427. 25056061 10.1038/nature13595 PMC4112379

[brb33429-bib-0009] Pierce, B. L. , Ahsan, H. , & Vanderweele, T. J. (2011). Power and instrument strength requirements for Mendelian randomization studies using multiple genetic variants. International Journal of Epidemiology, 40(3), 740–752. 10.1093/ije/dyq151 20813862 PMC3147064

[brb33429-bib-0007] Pye, S. R. , Marshall, T. , Gaffney, K. , Silman, A. J. , Symmons, D. P. M , & O'neill, T. W. (2010). Influence of arthritis and non‐arthritis related factors on areal bone mineral density (BMDa) in women with longstanding inflammatory polyarthritis: A primary care based inception cohort. BMC Musculoskeletal Disorders, 11, 106. 10.1186/1471-2474-11-106 20509941 PMC2889849

[brb33429-bib-0044] Ren, K. , Wan, H. , Kaper, H. J. , & Sharma, P. K. (2022). Dopamine‐conjugated hyaluronic acid delivered via intra‐articular injection provides articular cartilage lubrication and protection. Journal of Colloid and Interface Science, 619, 207–218. 10.1016/j.jcis.2022.03.119 35397456

[brb33429-bib-0036] Rødevand, L. , Bahrami, S. , Frei, O. , Chu, Y. , Shadrin, A. , O'connell, K. S. , Smeland, O. B. , Elvsåshagen, T. , Hindley, G. F. L. , Djurovic, S. , Dale, A. M. , Lagerberg, T. V. , Steen, N. E. , & Andreassen, O. A. (2021). Extensive bidirectional genetic overlap between bipolar disorder and cardiovascular disease phenotypes. Translational Psychiatry, 11(1), 407. 10.1038/s41398-021-01527-z 34301917 PMC8302675

[brb33429-bib-0043] Sanderson, E. , Glymour, M. M. , Holmes, M. V. , Kang, H. , Morrison, J. , Munafò, M. R. , Palmer, T. , Schooling, C. M. , Wallace, C. , Zhao, Q. , & Smith, G. D. (2022). Mendelian randomization. Nature Reviews Methods Primers, 2, Article 6. 10.1038/s43586-021-00092-5 PMC7614635 37325194

[brb33429-bib-0008] Sarkar, C. , Basu, B. , Chakroborty, D. , Dasgupta, P. S. , & Basu, S. (2010). The immunoregulatory role of dopamine: An update. Brain, Behavior, and Immunity, 24(4), 525–528. 10.1016/j.bbi.2009.10.015 19896530 PMC2856781

[brb33429-bib-0031] Schwartz, A. M. , Farley, K. X. , Guild, G. N. , & Bradbury, T. L. (2020). Projections and epidemiology of revision hip and knee arthroplasty in the United States to 2030. The Journal of Arthroplasty, 35(6s), S79–S85. 10.1016/j.arth.2020.02.030 32151524 PMC7239745

[brb33429-bib-0042] Stoica, C. I. , Nedelea, G. , Cotor, D. C. , Gherghe, M. , Georgescu, D. E. , Dragosloveanu, C. , & Dragosloveanu, S. (2022). The outcome of total knee arthroplasty for patients with psychiatric disorders: A single‐center retrospective study. Medicina, 58(9), 1277. 10.3390/medicina58091277 36143953 PMC9502460

[brb33429-bib-0024] Sugai, K. , Takeda‐Imai, F. , Michikawa, T. , Nakamura, T. , Takebayashi, T. , & Nishiwaki, Y. (2018). Association between knee pain, impaired function, and development of depressive symptoms. Journal of the American Geriatrics Society, 66(3), 570–576. 10.1111/jgs.15259 29441517

[brb33429-bib-0027] Tachmazidou, I. , Hatzikotoulas, K. , Southam, L. , Esparza‐Gordillo, J. , Haberland, V. , Zheng, J. , Johnson, T. , Koprulu, M. , Zengini, E. , Steinberg, J. , Wilkinson, J. M. , Bhatnagar, S. , Hoffman, J. D. , Buchan, N. , Süveges, D. , Yerges‐Armstrong, L. , Smith, G. D. , Gaunt, T. R. , Scott, R. A. , … Zeggini, E. (2019). Identification of new therapeutic targets for osteoarthritis through genome‐wide analyses of UK Biobank data. Nature Genetics, 51(2), 230–236. 10.1038/s41588-018-0327-1 30664745 PMC6400267

[brb33429-bib-0032] Ward, M. M. , & Dasgupta, A. (2020). Regional variation in rates of total knee arthroplasty among medicare beneficiaries. JAMA Network Open, 3(4), e203717. 10.1001/jamanetworkopen.2020.3717 32343352 PMC7189226

[brb33429-bib-0045] Xiang, S. , Wang, R. , Hua, L. , Song, J. , Qian, S. , Jin, Y. , Zhang, B. , & Ding, X. (2023). Assessment of bidirectional relationships between mental illness and rheumatoid arthritis: A two‐sample Mendelian randomization study. Journal of Clinical Medicine, 12(3), 944. 10.3390/jcm12030944 36769592 PMC9917759

[brb33429-bib-0014] Yan, Y. , Jiang, W. , Liu, L. , Wang, X. , Ding, C. , Tian, Z. , & Zhou, R. (2015). Dopamine controls systemic inflammation through inhibition of NLRP3 inflammasome. Cell, 160(1‐2), 62–73. 10.1016/j.cell.2014.11.047 25594175

[brb33429-bib-0021] Zheng, J. , Baird, D. , Borges, M.‐C. , Bowden, J. , Hemani, G. , Haycock, P. , Evans, D. M. , & Smith, G. D. (2017). Recent developments in Mendelian randomization studies. Current Epidemiology Reports, 4(4), 330–345. 10.1007/s40471-017-0128-6 29226067 PMC5711966

[brb33429-bib-0038] Zhong, G. , Long, H. , Chen, F. , & Yu, Y. (2021). Oxoglaucine mediates Ca^2+^ influx and activates autophagy to alleviate osteoarthritis through the TRPV5/calmodulin/CAMK‐II pathway. British Journal of Pharmacology, 178(15), 2931–2947. 10.1111/bph.15466 33786819

