# Supplementary material for: Bidirectional causal relationship between psychiatric disorders and osteoarthritis: A univariate and multivariate Mendelian randomization study
Source: Brain Behav. 2024 Feb 15;14(2):e3429. doi: 10.1002/brb3.3429 (PMC10869882; doi:10.1002/brb3.3429)
Supplement: Supplementary file 1 — Figure S1: Scatter plot regarding the causal effect of psychiatric disorders on OA. Scatter plot of bipolar disorder on KOA (A), HOA (B) and KHOA (C); major depression on KOA (D), HOA (E) and KHOA (F); schizophrenia on KOA (G), HOA (H) and KHOA (I). KOA, Knee OA; HOA, Hip OA; KHOA, Osteoarthritis of knee or hip. Figure S2: Leave‐one‐out analysis for the causal effect of psychiatric disorders on OA. Leave‐one‐out analysis of bipolar disorder on KOA (A), HOA (B) and KHOA (C); major depression on KOA (D), HOA (E) and KHOA (F); schizophrenia on KOA (G), HOA (H) and KHOA (I). KOA, Knee OA; HOA, Hip OA; KHOA, Osteoarthritis of knee or hip. Figure S3: Forest plot for the causal effects of psychiatric disorders on OA. (A) Forest plot for the causal effects of bipolar disorder on KOA (A), HOA (B) and KHOA (C); major depression on KOA (D), HOA (E) and KHOA (F); schizophrenia on KOA (G), HOA (H) and KHOA (I). KOA, Knee OA; HOA, Hip OA; KHOA, Osteoarthritis of knee or hip. Figure S4: Scatter plot regarding the causal effect of OA on psychiatric disorders in the inverse MR Analysis. Scatter plots of causal effects of KOA on bipolar disorder (A), major depression (B), and schizophrenia (C); HOA on bipolar disorder (D), major depression (E), and schizophrenia (F); KHOA on bipolar disorder (G), major depression (H), and schizophrenia (I). KOA, Knee OA; HOA, Hip OA; KHOA, Osteoarthritis of knee or hip. Figure S5: Leave‐one‐out analysis for the causal effect of OA on psychiatric disorders in the inverse MR Analysis. Leave‐one‐out analysis for the causal effect of KOA on bipolar disorder (A), major depression (B), and schizophrenia (C); HOA on bipolar disorder (D), major depression (E), and schizophrenia (F); KHOA on bipolar disorder (G), major depression (H), and schizophrenia (I). KOA, Knee OA; HOA, Hip OA; KHOA, Osteoarthritis of knee or hip. Figure S6: Forest plot for the causal effects of OA on psychiatric disorders in the inverse MR Analysis. Forest plot for the overall causal e [file BRB3-14-e3429-s001.pdf]

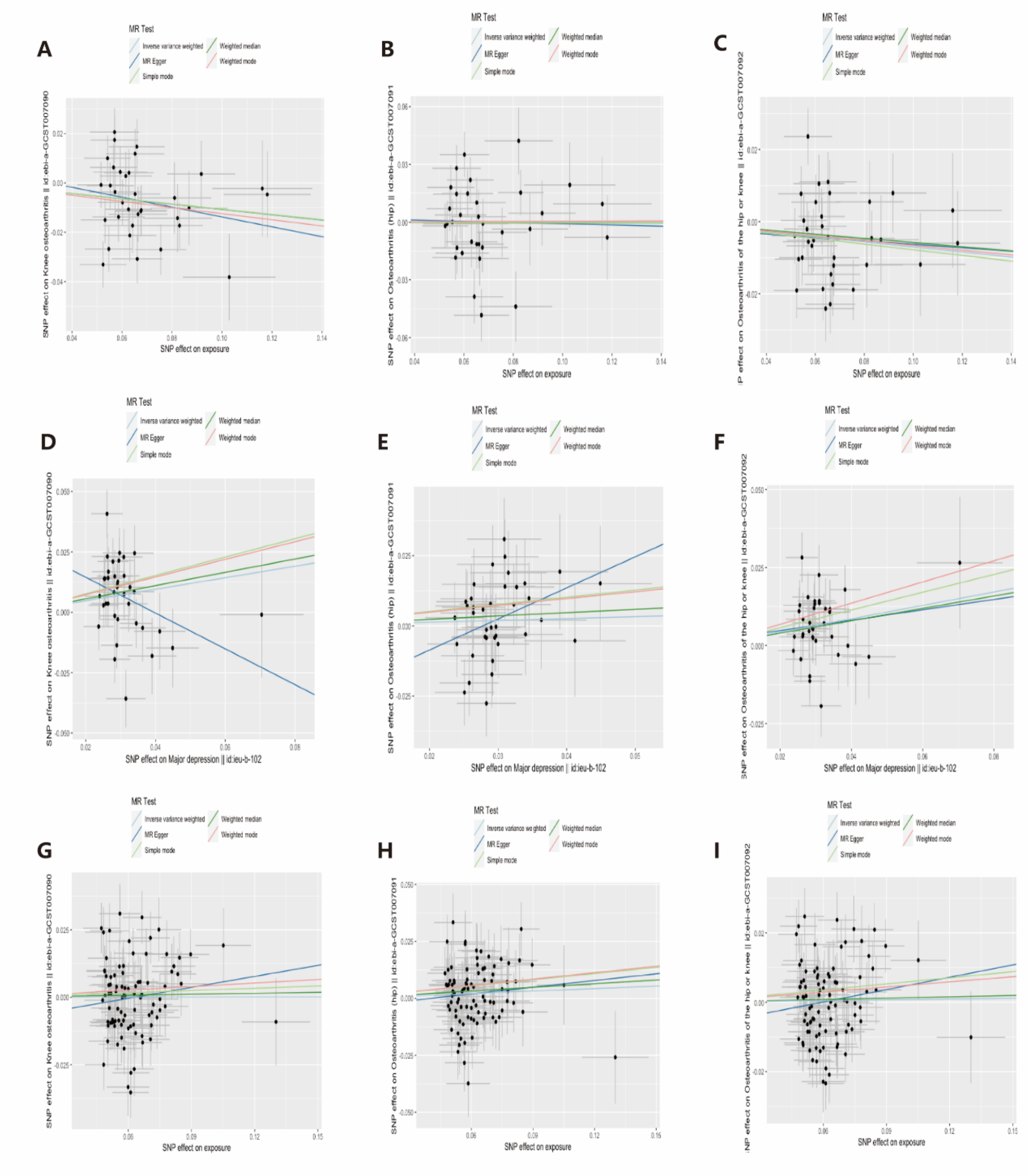

Figure S1: Scatter plot regarding the causal effect of psychiatric disorders on OA. Scatter plot of bipolar disorder on KOA (A), HOA (B) and KHOA (C); major depression on KOA (D), HOA (E) and KHOA (F); schizophrenia on KOA (G), HOA (H) and KHOA (I). KOA, Knee OA; HOA, Hip OA; KHOA, Osteoarthritis of knee or hip.

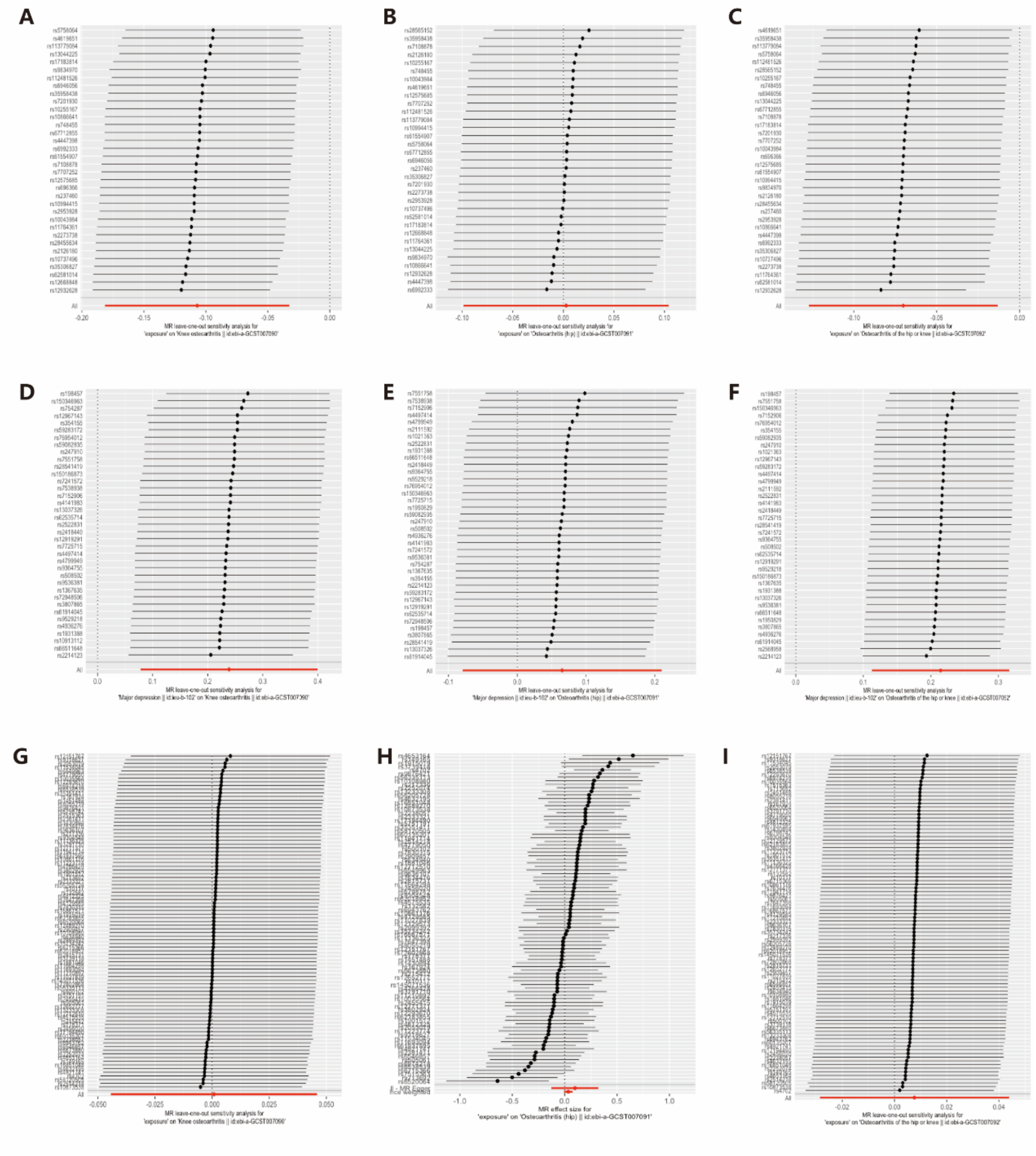

Figure S2: Leave-one-out analysis for the causal effect of psychiatric disorders on OA. Leave-one-out analysis of bipolar disorder on KOA (A), HOA (B) and KHOA (C); major depression on KOA (D), HOA (E) and KHOA (F); schizophrenia on KOA (G), HOA (H) and KHOA (I). KOA, Knee OA; HOA, Hip OA; KHOA, Osteoarthritis of knee or hip.

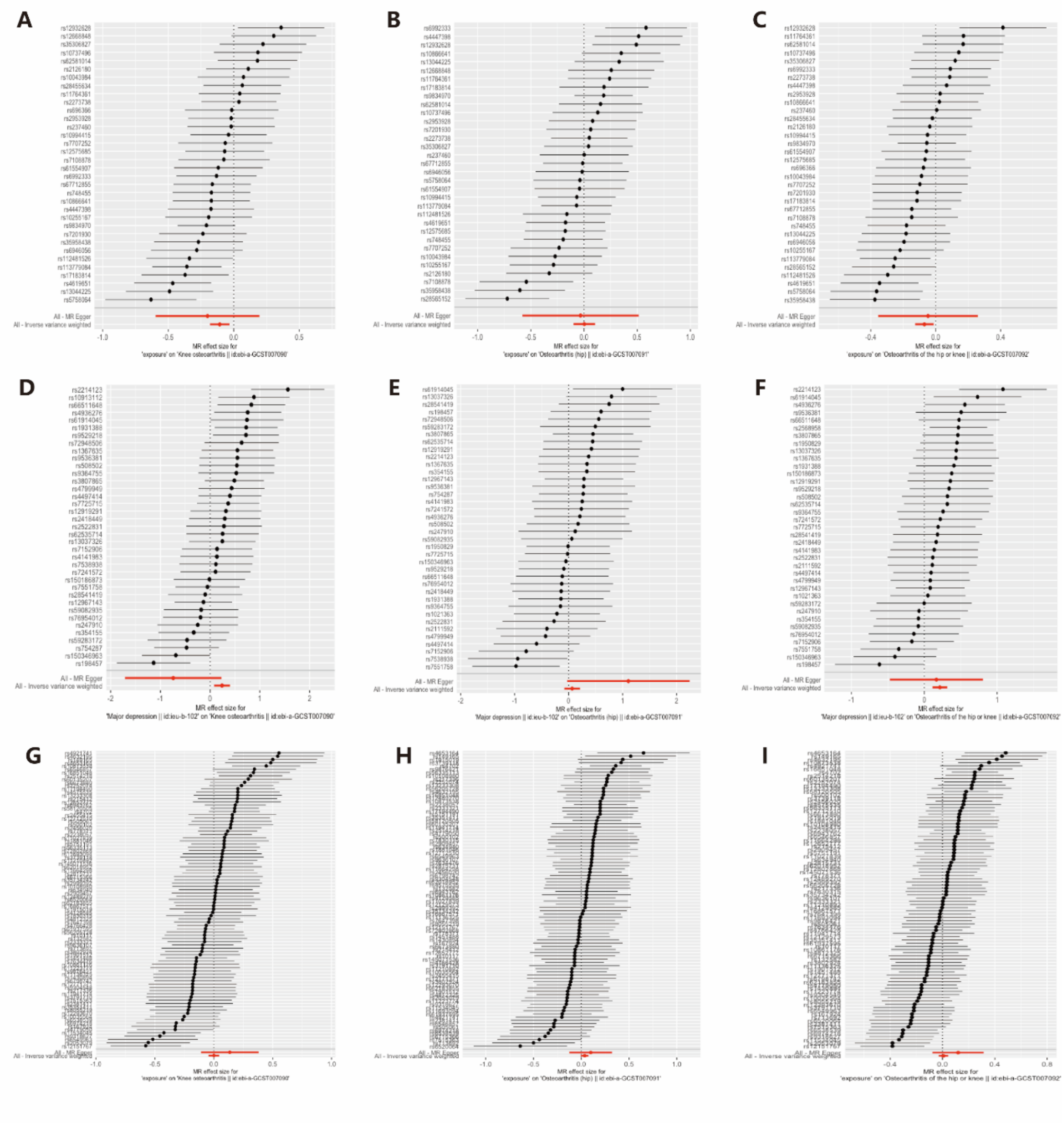

Figure S3: Forest plot for the causal effects of psychiatric disorders on OA. (A) Forest plot for the causal effects of bipolar disorder on KOA (A), HOA (B) and KHOA (C); major depression on KOA (D), HOA (E) and KHOA (F); schizophrenia on KOA (G), HOA (H) and KHOA (I). KOA, Knee OA; HOA, Hip OA; KHOA, Osteoarthritis of knee or hip.

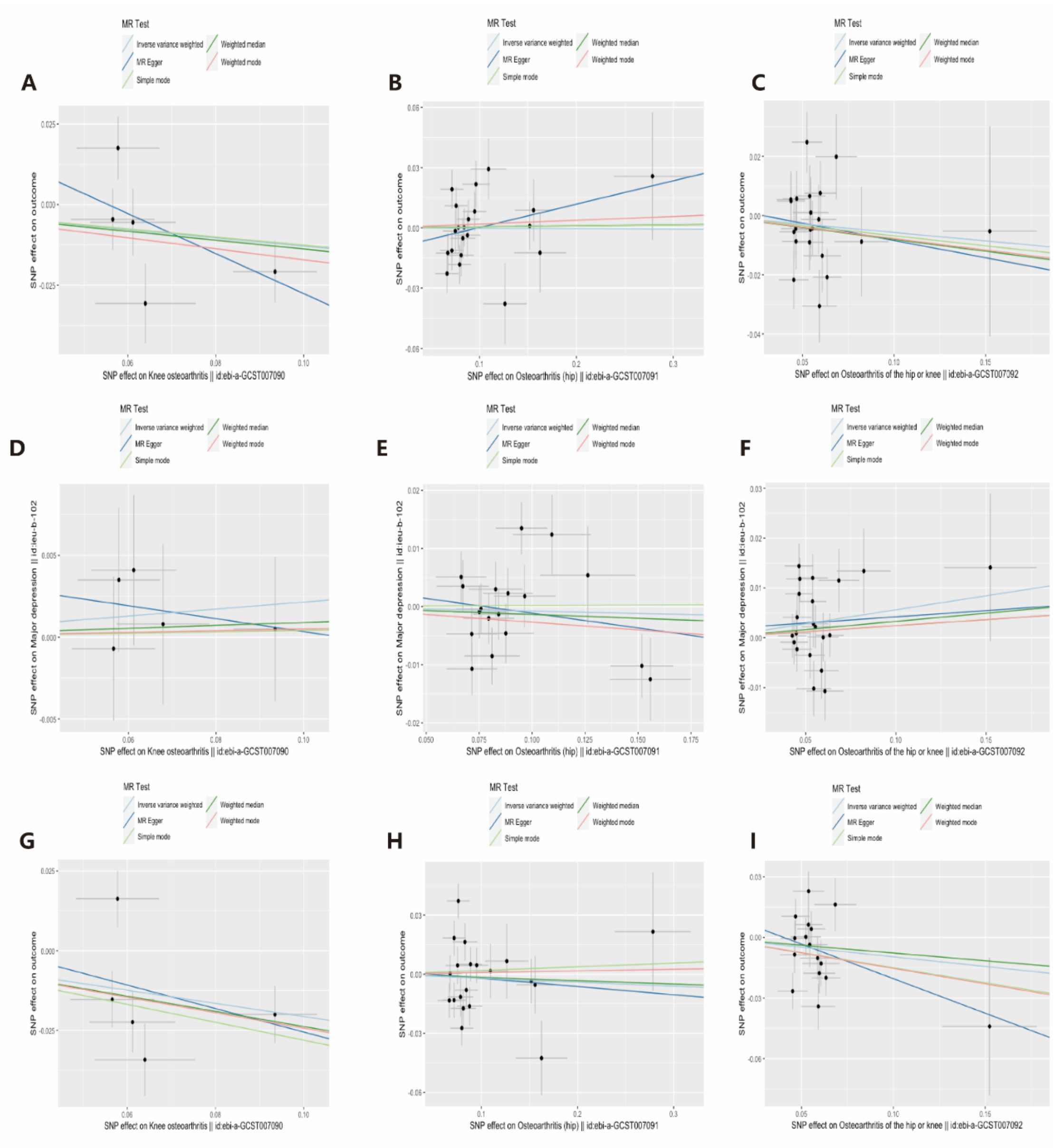

Figure S4: Scatter plot regarding the causal effect of OA on psychiatric disorders in the inverse MR Analysis. Scatter plots of causal effects of KOA on bipolar disorder (A), major depression (B), and schizophrenia (C); HOA on bipolar disorder (D), major depression (E), and schizophrenia (F); KHOA on bipolar disorder (G), major depression (H), and schizophrenia (I). KOA, Knee OA; HOA, Hip OA; KHOA, Osteoarthritis of knee or hip.

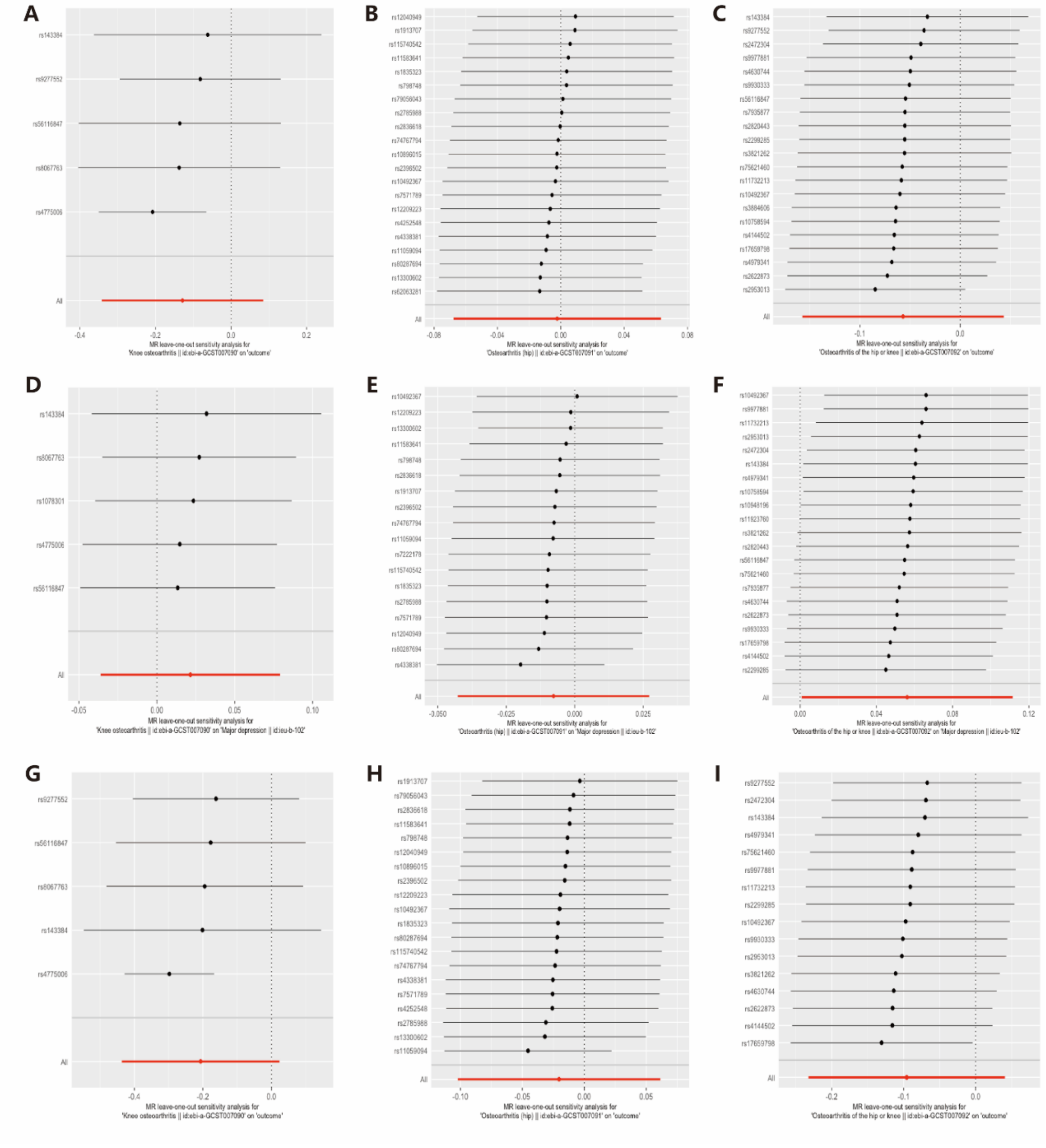

Figure S5: Leave-one-out analysis for the causal effect of OA on psychiatric disorders in the inverse MR Analysis. Leave-one-out analysis for the causal effect of KOA on bipolar disorder (A), major depression (B), and schizophrenia (C); HOA on bipolar disorder (D), major depression (E), and schizophrenia (F); KHOA on bipolar disorder (G), major depression (H), and schizophrenia (I). KOA, Knee OA; HOA, Hip OA; KHOA, Osteoarthritis of knee or hip.

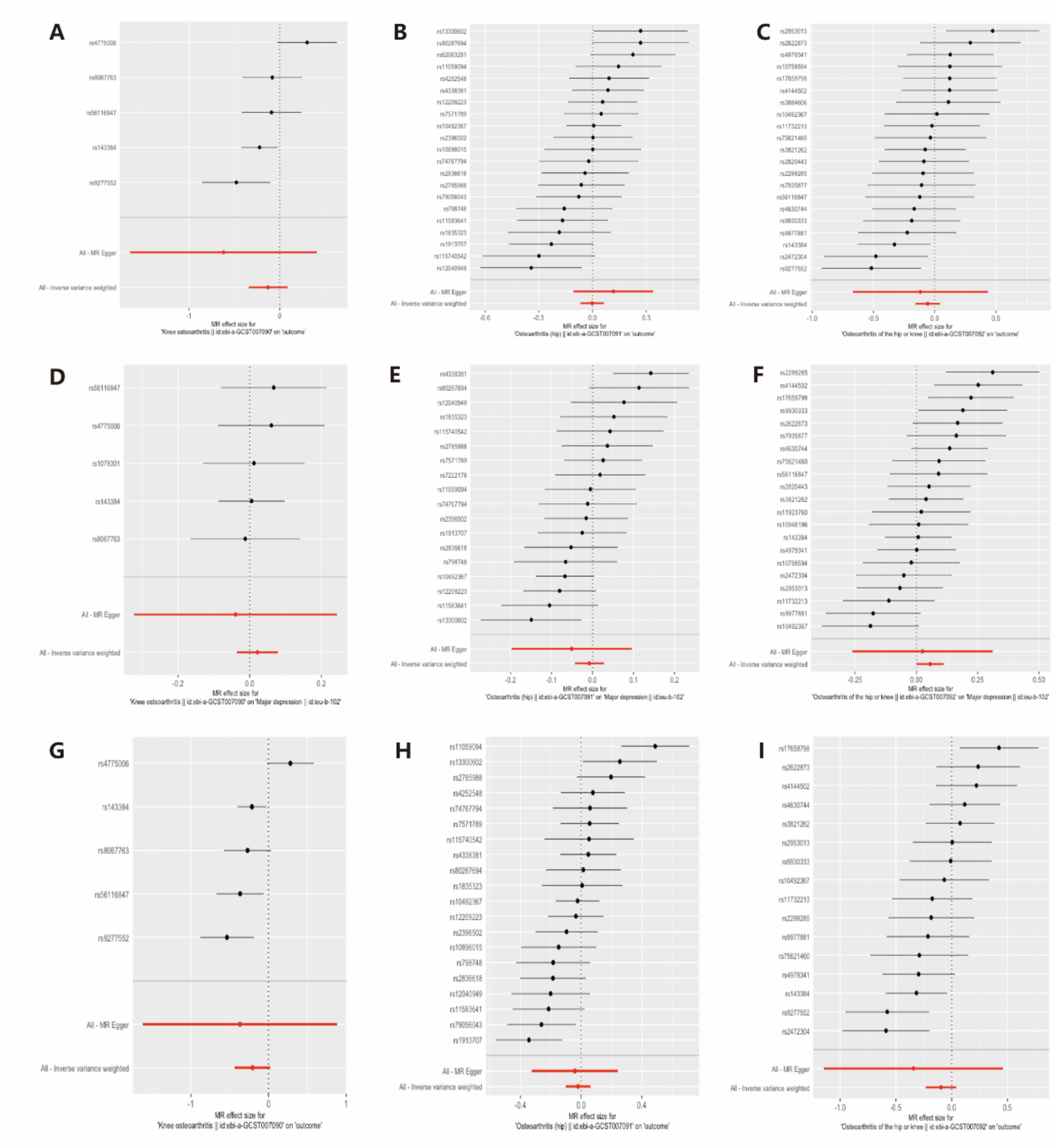

Figure S6: Forest plot for the causal effects of OA on psychiatric disorders in the inverse MR Analysis. Forest plot for the overall causal effects of KOA on bipolar disorder (A), major depression (B), and schizophrenia (C); HOA on bipolar disorder (D), major depression (E), and schizophrenia (F); KHOA on bipolar disorder (G), major depression (H), and schizophrenia (I). KOA, Knee OA; HOA, Hip OA; KHOA, Osteoarthritis of knee or hip.
