# Supplementary material for: Bidirectional causal relationship between psychiatric disorders and osteoarthritis: A univariate and multivariate Mendelian randomization study
Source: Brain Behav. 2024 Feb 15;14(2):e3429. doi: 10.1002/brb3.3429 (PMC10869882; doi:10.1002/brb3.3429)
Supplement: Supplementary file 2 — Table S1. 34 valid IVs used for MR analysis of bipolar disorder on KOA Table S2. 35 valid IVs used for MR analysis of bipolar disorder on HOA Table S3. 34 valid IVs used for MR analysis of bipolar disorder on KHOA Table S4. 5 valid IVs used for MR analysis of KOA on bipolar disorder Table S5. 21valid IVs used for MR analysis of HOA on bipolar disorder Table S6. 21 valid IVs used for MR analysis of KHOA on bipolar disorder Table S7. 37 valid IVs used for MR analysis of major depression on KOA Table S8. 38 valid IVs used for MR analysis of major depression on HOA Table S9. 37 valid IVs used for MR analysis of major depression on KHOA Table S10. 5 valid IVs used for MR analysis of KOA on major depression Table S11. 18 valid IVs used for MR analysis of HOA on major depression Table S12. 21 valid IVs used for MR analysis of KHOA on major depression Table S13. 91 valid IVs used for MR analysis of schizophrenia on KOA Table S14. 92 valid IVs used for MR analysis of schizophrenia on HOA Table S15. 92 valid IVs used for MR analysis of schizophrenia on KHOA Table S16. 5 valid IVs used for MR analysis of KOA on schizophrenia Table S17. 15 valid IVs used for MR analysis of HOA on schizophrenia Table S18. 16 valid IVs used for MR analysis of KHOA on schizophrenia. [file BRB3-14-e3429-s002.docx]

**Table S1. 34 valid IVs used for MR analysis of bipolar disorder on KOA**

| SNPs | Effect  allele | Non-effect  allele | Beta | Effect allele frequency | SE | P-value | F Statistics |
| --- | --- | --- | --- | --- | --- | --- | --- |
| rs10043984 | C | T | -0.0593 | 0.7423 | 0.0108 | 3.71E-08 | 11.82229 |
| rs10255167 | G | A | -0.0664 | 0.2217 | 0.0118 | 1.60E-08 | 10.76646 |
| rs10737496 | C | T | 0.054204 | 0.4613 | 0.0094 | 7.17E-09 | 16.38196 |
| rs10866641 | T | C | 0.062599 | 0.5625 | 0.0094 | 2.79E-11 | 21.5963 |
| rs10994415 | T | C | -0.1181 | 0.9245 | 0.0174 | 1.14E-11 | 14.5428 |
| rs112481526 | A | G | -0.0631 | 0.7375 | 0.0105 | 1.86E-09 | 16.05035 |
| rs113779084 | G | A | -0.0755 | 0.7009 | 0.0102 | 1.42E-13 | 21.98521 |
| rs11764361 | A | G | 0.0615 | 0.6579 | 0.0104 | 3.47E-09 | 15.17322 |
| rs12575685 | G | A | -0.0652 | 0.689 | 0.0101 | 1.24E-10 | 16.33939 |
| rs12668848 | G | A | 0.057004 | 0.5644 | 0.0095 | 1.90E-09 | 17.64322 |
| rs12932628 | G | T | -0.057 | 0.5301 | 0.0098 | 6.71E-09 | 16.89037 |
| rs13044225 | A | G | -0.0547 | 0.5318 | 0.0095 | 8.50E-09 | 16.44948 |
| rs17183814 | G | A | 0.102899 | 0.9243 | 0.0185 | 2.68E-08 | 14.89889 |
| rs2126180 | G | A | -0.0566 | 0.5338 | 0.0094 | 1.62E-09 | 16.87779 |
| rs2273738 | C | T | -0.0917 | 0.8578 | 0.0136 | 1.63E-11 | 21.2816 |
| rs237460 | C | T | -0.0553 | 0.5817 | 0.0094 | 4.25E-09 | 16.11272 |
| rs28455634 | G | A | 0.062796 | 0.6186 | 0.0099 | 2.63E-10 | 12.479 |
| rs2953928 | G | A | -0.1161 | 0.939 | 0.02 | 6.25E-09 | 14.13672 |
| rs35306827 | G | A | 0.0659 | 0.7757 | 0.0112 | 3.56E-09 | 11.77299 |
| rs35958438 | G | A | 0.064204 | 0.7738 | 0.0117 | 3.83E-08 | 17.53062 |
| rs4447398 | A | C | 0.082197 | 0.1328 | 0.0138 | 2.61E-09 | 16.44277 |
| rs4619651 | G | A | 0.066097 | 0.6801 | 0.0101 | 4.78E-11 | 17.1851 |
| rs5758064 | T | C | 0.052403 | 0.5228 | 0.0093 | 2.01E-08 | 15.51056 |
| rs61554907 | G | T | -0.0868 | 0.8909 | 0.0154 | 1.64E-08 | 17.48455 |
| rs62581014 | C | T | -0.0652 | 0.6271 | 0.0117 | 2.77E-08 | 14.00452 |
| rs67712855 | T | G | 0.067696 | 0.7116 | 0.0103 | 4.22E-11 | 16.52269 |
| rs6946056 | A | C | -0.0532 | 0.4029 | 0.0097 | 3.66E-08 | 14.08102 |
| rs696366 | C | A | 0.051596 | 0.5332 | 0.0094 | 4.46E-08 | 14.98902 |
| rs6992333 | A | G | -0.0602 | 0.5701 | 0.01 | 1.62E-09 | 17.80562 |
| rs7108878 | T | G | -0.081 | 0.8824 | 0.0147 | 3.61E-08 | 15.01865 |
| rs7201930 | T | C | -0.0586 | 0.6785 | 0.0104 | 1.89E-08 | 14.31971 |
| rs748455 | T | C | 0.0675 | 0.7067 | 0.0103 | 5.01E-11 | 17.96925 |
| rs7707252 | A | G | -0.05719 | 0.7352 | 0.0104 | 3.64E-08 | 12.65442 |
| rs9834970 | T | C | -0.083 | 0.5022 | 0.0093 | 6.63E-19 | 38.78997 |

**Table S2. 35 valid IVs used for MR analysis of bipolar disorder on HOA**

| SNPs | Effect  allele | Non-effect  allele | Beta | Effect allele frequency | SE | P-value | F Statistics |
| --- | --- | --- | --- | --- | --- | --- | --- |
| rs10043984 | C | T | -0.0593 | 0.7427 | 0.0108 | 3.71E-08 | 11.82231 |
| rs10255167 | G | A | -0.0664 | 0.2217 | 0.0118 | 1.60E-08 | 10.76647 |
| rs10737496 | C | T | 0.054204 | 0.4614 | 0.0094 | 7.17E-09 | 16.382 |
| rs10866641 | T | C | 0.062599 | 0.5629 | 0.0094 | 2.79E-11 | 21.59636 |
| rs10994415 | T | C | -0.1181 | 0.9244 | 0.0174 | 1.14E-11 | 16.45428 |
| rs112481526 | A | G | -0.0631 | 0.7373 | 0.0105 | 1.86E-09 | 16.05038 |
| rs113779084 | G | A | -0.0755 | 0.7004 | 0.0102 | 1.42E-13 | 21.98528 |
| rs11764361 | A | G | 0.0615 | 0.658 | 0.0104 | 3.47E-09 | 15.17325 |
| rs12575685 | G | A | -0.0652 | 0.6894 | 0.0101 | 1.24E-10 | 16.33942 |
| rs12668848 | G | A | 0.057004 | 0.5641 | 0.0095 | 1.90E-09 | 17.54807 |
| rs12932628 | G | T | -0.057 | 0.5306 | 0.0098 | 6.71E-09 | 16.57975 |
| rs13044225 | A | G | -0.0547 | 0.5309 | 0.0095 | 8.50E-09 | 16.54968 |
| rs17183814 | G | A | 0.102899 | 0.9244 | 0.0185 | 2.68E-08 | 14.33462 |
| rs2126180 | G | A | -0.0566 | 0.535 | 0.0094 | 1.62E-09 | 17.4616 |
| rs2273738 | C | T | -0.0917 | 0.8577 | 0.0136 | 1.63E-11 | 18.62363 |
| rs237460 | C | T | -0.0553 | 0.5816 | 0.0094 | 4.25E-09 | 16.20192 |
| rs28565152 | G | A | -0.0671 | 0.7483 | 0.0112 | 1.96E-09 | 11.13301 |
| rs2953928 | G | A | -0.1161 | 0.9392 | 0.02 | 6.25E-09 | 14.13672 |
| rs35306827 | G | A | 0.0659 | 0.7757 | 0.0112 | 3.56E-09 | 11.77301 |
| rs35958438 | G | A | 0.064204 | 0.7737 | 0.0117 | 3.83E-08 | 17.53062 |
| rs4447398 | A | C | 0.082197 | 0.1334 | 0.0138 | 2.61E-09 | 16.44278 |
| rs4619651 | G | A | 0.066097 | 0.6807 | 0.0101 | 4.78E-11 | 17.18514 |
| rs5758064 | T | C | 0.052403 | 0.5235 | 0.0093 | 2.01E-08 | 15.51059 |
| rs61554907 | G | T | -0.0868 | 0.891 | 0.0154 | 1.64E-08 | 17.48455 |
| rs62581014 | C | T | -0.0652 | 0.6271 | 0.0117 | 2.77E-08 | 14.00454 |
| rs67712855 | T | G | 0.067696 | 0.7118 | 0.0103 | 4.22E-11 | 16.52273 |
| rs6946056 | A | C | -0.0532 | 0.403 | 0.0097 | 3.66E-08 | 14.08104 |
| rs6992333 | A | G | -0.0602 | 0.5695 | 0.01 | 1.62E-09 | 17.80566 |
| rs7108878 | T | G | -0.081 | 0.883 | 0.0147 | 3.61E-08 | 15.01865 |
| rs7201930 | T | C | -0.0586 | 0.6782 | 0.0104 | 1.89E-08 | 14.31974 |
| rs748455 | T | C | 0.0675 | 0.7065 | 0.0103 | 5.01E-11 | 17.96929 |
| rs7707252 | A | G | -0.05719 | 0.7357 | 0.0104 | 3.64E-08 | 12.65444 |
| rs9834970 | T | C | -0.083 | 0.5018 | 0.0093 | 6.63E-19 | 38.79018 |

**Table S3. 34 valid IVs used for MR analysis of bipolar disorder on KHOA**

| SNPs | Effect  allele | Non-effect  allele | Beta | Effect allele frequency | SE | P-value | F Statistics |
| --- | --- | --- | --- | --- | --- | --- | --- |
| rs10043984 | C | T | -0.0593 | 0.7423 | 0.0108 | 3.71E-08 | 11.82229 |
| rs10255167 | G | A | -0.0664 | 0.2217 | 0.0118 | 1.60E-08 | 10.76646 |
| rs10737496 | C | T | 0.054204 | 0.4613 | 0.0094 | 7.17E-09 | 16.38196 |
| rs10866641 | T | C | 0.062599 | 0.5625 | 0.0094 | 2.79E-11 | 21.5963 |
| rs10994415 | T | C | -0.1181 | 0.9245 | 0.0174 | 1.14E-11 | 14.5428 |
| rs112481526 | A | G | -0.0631 | 0.7375 | 0.0105 | 1.86E-09 | 16.05035 |
| rs113779084 | G | A | -0.0755 | 0.7009 | 0.0102 | 1.42E-13 | 21.98521 |
| rs11764361 | A | G | 0.0615 | 0.6579 | 0.0104 | 3.47E-09 | 15.17322 |
| rs12575685 | G | A | -0.0652 | 0.689 | 0.0101 | 1.24E-10 | 16.33939 |
| rs12668848 | G | A | 0.057004 | 0.5644 | 0.0095 | 1.90E-09 | 17.64322 |
| rs12932628 | G | T | -0.057 | 0.5301 | 0.0098 | 6.71E-09 | 16.89037 |
| rs13044225 | A | G | -0.0547 | 0.5318 | 0.0095 | 8.50E-09 | 16.44948 |
| rs17183814 | G | A | 0.102899 | 0.9243 | 0.0185 | 2.68E-08 | 14.89889 |
| rs2126180 | G | A | -0.0566 | 0.5338 | 0.0094 | 1.62E-09 | 16.87779 |
| rs2273738 | C | T | -0.0917 | 0.8578 | 0.0136 | 1.63E-11 | 21.2816 |
| rs237460 | C | T | -0.0553 | 0.5817 | 0.0094 | 4.25E-09 | 16.11272 |
| rs28455634 | G | A | 0.062796 | 0.6186 | 0.0099 | 2.63E-10 | 12.479 |
| rs2953928 | G | A | -0.1161 | 0.939 | 0.02 | 6.25E-09 | 14.13672 |
| rs35306827 | G | A | 0.0659 | 0.7757 | 0.0112 | 3.56E-09 | 11.77299 |
| rs35958438 | G | A | 0.064204 | 0.7738 | 0.0117 | 3.83E-08 | 17.53062 |
| rs4447398 | A | C | 0.082197 | 0.1328 | 0.0138 | 2.61E-09 | 16.44277 |
| rs4619651 | G | A | 0.066097 | 0.6801 | 0.0101 | 4.78E-11 | 17.1851 |
| rs5758064 | T | C | 0.052403 | 0.5228 | 0.0093 | 2.01E-08 | 15.51056 |
| rs61554907 | G | T | -0.0868 | 0.8909 | 0.0154 | 1.64E-08 | 17.48455 |
| rs62581014 | C | T | -0.0652 | 0.6271 | 0.0117 | 2.77E-08 | 14.00452 |
| rs67712855 | T | G | 0.067696 | 0.7116 | 0.0103 | 4.22E-11 | 16.52269 |
| rs6946056 | A | C | -0.0532 | 0.4029 | 0.0097 | 3.66E-08 | 14.08102 |
| rs696366 | C | A | 0.051596 | 0.5332 | 0.0094 | 4.46E-08 | 14.98902 |
| rs6992333 | A | G | -0.0602 | 0.5701 | 0.01 | 1.62E-09 | 17.80562 |
| rs7108878 | T | G | -0.081 | 0.8824 | 0.0147 | 3.61E-08 | 15.01865 |
| rs7201930 | T | C | -0.0586 | 0.6785 | 0.0104 | 1.89E-08 | 14.31971 |
| rs748455 | T | C | 0.0675 | 0.7067 | 0.0103 | 5.01E-11 | 17.96925 |
| rs7707252 | A | G | -0.05719 | 0.7352 | 0.0104 | 3.64E-08 | 12.65442 |
| rs9834970 | T | C | -0.083 | 0.5022 | 0.0093 | 6.63E-19 | 38.78997 |

**Table S4. 5 valid IVs used for MR analysis of KOA on bipolar disorder**

| SNPs | Effect  allele | Non-effect  allele | Beta | Effect allele frequency | SE | P-value | F Statistics |
| --- | --- | --- | --- | --- | --- | --- | --- |
| rs143384 | G | A | -0.0935 | 0.4034 | 0.0095 | 4.77E-23 | 46.92137 |
| rs4775006 | A | C | 0.0578 | 0.4114 | 0.0094 | 8.40E-10 | 18.89779 |
| rs56116847 | A | G | 0.0612 | 0.3563 | 0.0097 | 3.19E-10 | 19.43842 |
| rs8067763 | A | G | -0.0566 | 0.5936 | 0.0095 | 2.39E-09 | 17.12959 |
| rs9277552 | T | C | -0.064 | 0.2105 | 0.0114 | 1.97E-08 | 11.25179 |

**Table S5. 21valid IVs used for MR analysis of HOA on bipolar disorder**

| SNPs | Effect  allele | Non-effect  allele | Beta | Effect allele frequency | SE | P-value | F Statistics |
| --- | --- | --- | --- | --- | --- | --- | --- |
| rs10492367 | T | G | 0.1518 | 0.19 | 0.0148 | 1.25E-24 | 35.16401 |
| rs10896015 | A | G | -0.0782 | 0.2695 | 0.0132 | 2.74E-09 | 17.54186 |
| rs11059094 | T | C | 0.0759 | 0.4777 | 0.0117 | 7.38E-11 | 13.82144 |
| rs115740542 | C | T | 0.1263 | 0.0741 | 0.0224 | 1.60E-08 | 14.53157 |
| rs11583641 | T | C | -0.0811 | 0.2764 | 0.0131 | 5.57E-10 | 15.0273 |
| rs12040949 | T | C | -0.0665 | 0.3843 | 0.012 | 2.83E-08 | 13.32541 |
| rs12209223 | A | C | 0.1558 | 0.1032 | 0.0191 | 3.88E-16 | 29.79577 |
| rs13300602 | G | A | 0.0716 | 0.4508 | 0.0119 | 1.65E-09 | 18.52896 |
| rs1835323 | T | C | -0.0673 | 0.3428 | 0.0123 | 4.56E-08 | 12.27563 |
| rs1913707 | G | A | -0.0795 | 0.3877 | 0.012 | 2.96E-11 | 18.53939 |
| rs2396502 | C | A | 0.0842 | 0.6018 | 0.012 | 2.12E-12 | 24.59547 |
| rs2785988 | A | C | 0.0828 | 0.2988 | 0.0127 | 7.30E-11 | 21.17613 |
| rs2836618 | A | G | 0.0876 | 0.2613 | 0.0132 | 3.20E-11 | 21.878 |
| rs4252548 | T | C | 0.2785 | 0.022 | 0.0396 | 1.96E-12 | 24.72167 |
| rs4338381 | G | A | -0.095 | 0.3681 | 0.0121 | 4.37E-15 | 25.27637 |
| rs62063281 | G | A | 0.0964 | 0.2229 | 0.014 | 5.30E-12 | 22.31474 |
| rs74767794 | G | A | -0.0751 | 0.3171 | 0.0126 | 2.56E-09 | 12.75449 |
| rs7571789 | C | T | -0.0886 | 0.5239 | 0.0117 | 3.26E-14 | 15.49941 |
| rs79056043 | G | A | 0.1625 | 0.0503 | 0.0268 | 1.33E-09 | 11.63214 |
| rs798748 | C | T | 0.0715 | 0.6183 | 0.012 | 2.50E-09 | 17.33493 |
| rs80287694 | G | A | 0.1093 | 0.1131 | 0.0184 | 2.66E-09 | 10.34534 |

**Table S6. 21 valid IVs used for MR analysis of KHOA on bipolar disorder**

| SNPs | Effect  allele | Non-effect  allele | Beta | Effect allele frequency | SE | P-value | F Statistics |
| --- | --- | --- | --- | --- | --- | --- | --- |
| rs10492367 | T | G | 0.0545 | 0.1895 | 0.0097 | 1.96E-08 | 10.55116 |
| rs10758594 | G | A | 0.0436 | 0.5844 | 0.0077 | 1.69E-08 | 16.02503 |
| rs11732213 | C | T | -0.0588 | 0.1945 | 0.0096 | 8.81E-10 | 12.32116 |
| rs143384 | G | A | -0.0634 | 0.4035 | 0.0077 | 2.42E-16 | 30.98954 |
| rs17659798 | C | A | -0.0539 | 0.2869 | 0.0085 | 2.06E-10 | 15.766 |
| rs2299285 | A | G | 0.0463 | 0.3434 | 0.008 | 7.57E-09 | 14.5339 |
| rs2472304 | A | G | 0.0452 | 0.668 | 0.0081 | 2.03E-08 | 13.94361 |
| rs2622873 | C | T | -0.0684 | 0.129 | 0.0113 | 1.58E-09 | 18.63215 |
| rs2820443 | C | T | 0.0543 | 0.2985 | 0.0083 | 6.01E-11 | 17.5498 |
| rs2953013 | A | C | -0.0524 | 0.7048 | 0.0083 | 3.07E-10 | 16.83555 |
| rs3821262 | G | A | -0.0554 | 0.474 | 0.0076 | 3.52E-13 | 26.54522 |
| rs3884606 | A | G | -0.0437 | 0.5119 | 0.0076 | 8.25E-09 | 16.47111 |
| rs4144502 | A | G | 0.0468 | 0.5118 | 0.0076 | 9.48E-10 | 18.83681 |
| rs4630744 | G | A | -0.0538 | 0.4916 | 0.0076 | 2.10E-12 | 25.04691 |
| rs4979341 | T | C | 0.0597 | 0.2697 | 0.0086 | 3.35E-12 | 19.75982 |
| rs56116847 | A | G | 0.0453 | 0.3564 | 0.008 | 1.28E-08 | 15.09042 |
| rs75621460 | A | G | 0.1523 | 0.0267 | 0.0256 | 2.88E-09 | 12.74424 |
| rs7935877 | T | C | -0.0822 | 0.071 | 0.0149 | 3.41E-08 | 12.91869 |
| rs9277552 | T | C | -0.0592 | 0.2098 | 0.0093 | 2.37E-10 | 12.82037 |
| rs9930333 | G | T | 0.0464 | 0.4234 | 0.0077 | 1.51E-09 | 17.73072 |
| rs9977881 | C | T | 0.0607 | 0.1695 | 0.0102 | 2.54E-09 | 19.37922 |

**Table S7. 37 valid IVs used for MR analysis of major depression on KOA**

| SNPs | Effect  allele | Non-effect  allele | Beta | Effect allele frequency | SE | P-value | F Statistics |
| --- | --- | --- | --- | --- | --- | --- | --- |
| rs10913112 | T | C | -0.0262 | 0.378 | 0.0045 | 4.53E-09 | 15.78088 |
| rs12919291 | C | G | 0.0327 | 0.1884 | 0.0055 | 3.09E-09 | 10.51629 |
| rs12967143 | C | G | -0.0345 | 0.7012 | 0.0047 | 2.53E-13 | 23.57368 |
| rs13037326 | T | C | 0.031 | 0.2597 | 0.0049 | 2.40E-10 | 14.05998 |
| rs1367635 | C | T | 0.0253 | 0.5148 | 0.0043 | 4.35E-09 | 17.26542 |
| rs150186873 | C | A | 0.0704 | 0.0327 | 0.012 | 4.51E-09 | 16.88215 |
| rs150346963 | T | C | 0.0283 | 0.4118 | 0.0044 | 1.16E-10 | 20.07455 |
| rs1931388 | G | A | -0.0295 | 0.4042 | 0.0044 | 1.68E-11 | 22.14642 |
| rs198457 | T | C | -0.0315 | 0.1886 | 0.0056 | 1.90E-08 | 15.65946 |
| rs2214123 | G | A | -0.0261 | 0.6466 | 0.0045 | 8.56E-09 | 10.34611 |
| rs2418449 | C | T | -0.0281 | 0.281 | 0.0048 | 4.25E-09 | 13.90249 |
| rs247910 | G | A | 0.0237 | 0.457 | 0.0043 | 4.71E-08 | 13.88547 |
| rs2522831 | C | T | 0.024 | 0.4739 | 0.0043 | 2.11E-08 | 11.75977 |
| rs28541419 | G | C | -0.0292 | 0.2308 | 0.0052 | 1.76E-08 | 15.35505 |
| rs354155 | C | G | -0.0449 | 0.0923 | 0.0075 | 1.75E-09 | 17.73798 |
| rs3807865 | A | G | 0.031 | 0.4105 | 0.0044 | 1.09E-12 | 22.97337 |
| rs4141983 | C | T | -0.0264 | 0.326 | 0.0046 | 9.69E-09 | 11.57025 |
| rs4497414 | C | T | 0.0291 | 0.44 | 0.0044 | 2.93E-11 | 17.22923 |
| rs4799949 | T | C | -0.0292 | 0.6684 | 0.0046 | 1.40E-10 | 19.8992 |
| rs4936276 | C | G | 0.0278 | 0.622 | 0.0044 | 3.57E-10 | 18.68692 |
| rs508502 | T | C | -0.0264 | 0.2992 | 0.0048 | 3.56E-08 | 15.00023 |
| rs59082935 | T | C | 0.0363 | 0.1342 | 0.0066 | 3.07E-08 | 14.08242 |
| rs59283172 | A | G | -0.039 | 0.1081 | 0.007 | 2.41E-08 | 14.75459 |
| rs61914045 | A | G | 0.0309 | 0.2034 | 0.0054 | 7.96E-09 | 12.68145 |
| rs62535714 | A | G | 0.0339 | 0.1639 | 0.0058 | 4.69E-09 | 18.29508 |
| rs66511648 | C | T | 0.0297 | 0.284 | 0.0048 | 6.03E-10 | 18.73378 |
| rs7152906 | C | T | 0.0258 | 0.5196 | 0.0043 | 1.87E-09 | 11.39002 |
| rs7241572 | A | G | 0.0323 | 0.2047 | 0.0054 | 2.43E-09 | 10.69501 |
| rs72948506 | A | G | 0.0265 | 0.2975 | 0.0047 | 1.71E-08 | 12.61061 |
| rs7538938 | C | T | 0.0251 | 0.5599 | 0.0043 | 7.29E-09 | 16.03604 |
| rs754287 | A | T | -0.0289 | 0.3664 | 0.0045 | 1.31E-10 | 11.17643 |
| rs7551758 | G | T | 0.0283 | 0.5329 | 0.0043 | 5.11E-11 | 21.39061 |
| rs76954012 | A | T | 0.0412 | 0.0931 | 0.0074 | 2.41E-08 | 17.96952 |
| rs7725715 | A | G | 0.029 | 0.5343 | 0.0043 | 1.61E-11 | 22.51118 |
| rs9364755 | G | A | 0.0283 | 0.2262 | 0.0051 | 3.49E-08 | 11.62373 |
| rs9529218 | T | C | -0.034 | 0.2031 | 0.0054 | 2.23E-10 | 11.79408 |
| rs9536381 | T | C | 0.0255 | 0.3259 | 0.0046 | 2.62E-08 | 14.73858 |

**Table S8. 38 valid IVs used for MR analysis of major depression on HOA**

| SNPs | Effect  allele | Non-effect  allele | Beta | Effect allele frequency | SE | P-value | F Statistics |
| --- | --- | --- | --- | --- | --- | --- | --- |
| rs1021363 | G | A | -0.03 | 0.6434 | 0.0045 | 2.29E-11 | 20.69079 |
| rs12919291 | C | G | 0.0327 | 0.1884 | 0.0055 | 3.09E-09 | 10.5163 |
| rs12967143 | C | G | -0.0345 | 0.7012 | 0.0047 | 2.53E-13 | 23.57371 |
| rs13037326 | T | C | 0.031 | 0.2597 | 0.0049 | 2.40E-10 | 14.05999 |
| rs1367635 | C | T | 0.0253 | 0.5148 | 0.0043 | 4.35E-09 | 17.26544 |
| rs150346963 | T | C | 0.0283 | 0.4118 | 0.0044 | 1.16E-10 | 20.07457 |
| rs1931388 | G | A | -0.0295 | 0.4042 | 0.0044 | 1.68E-11 | 22.14645 |
| rs1950829 | G | A | -0.0297 | 0.5173 | 0.0043 | 4.74E-12 | 23.61113 |
| rs198457 | T | C | -0.0315 | 0.1886 | 0.0056 | 1.90E-08 | 18.79062 |
| rs2111592 | A | G | 0.0263 | 0.3141 | 0.0046 | 1.35E-08 | 13.26044 |
| rs2214123 | G | A | -0.0261 | 0.6466 | 0.0045 | 8.56E-09 | 15.37654 |
| rs2418449 | C | T | -0.0281 | 0.281 | 0.0048 | 4.25E-09 | 12.93733 |
| rs247910 | G | A | 0.0237 | 0.457 | 0.0043 | 4.71E-08 | 14.79285 |
| rs2522831 | C | T | 0.024 | 0.4739 | 0.0043 | 2.11E-08 | 15.41762 |
| rs28541419 | G | C | -0.0292 | 0.2308 | 0.0052 | 1.76E-08 | 11.07667 |
| rs354155 | C | G | -0.0449 | 0.0923 | 0.0075 | 1.75E-09 | 15.92352 |
| rs3807865 | A | G | 0.031 | 0.4105 | 0.0044 | 1.09E-12 | 24.51372 |
| rs4141983 | C | T | -0.0264 | 0.326 | 0.0046 | 9.69E-09 | 15.41851 |
| rs4497414 | C | T | 0.0291 | 0.44 | 0.0044 | 2.93E-11 | 21.69007 |
| rs4799949 | T | C | -0.0292 | 0.6684 | 0.0046 | 1.40E-10 | 18.7589 |
| rs4936276 | C | G | 0.0278 | 0.622 | 0.0044 | 3.57E-10 | 18.97506 |
| rs508502 | T | C | -0.0264 | 0.2992 | 0.0048 | 3.56E-08 | 11.71558 |
| rs59082935 | T | C | 0.0363 | 0.1342 | 0.0066 | 3.07E-08 | 17.34517 |
| rs59283172 | A | G | -0.039 | 0.1081 | 0.007 | 2.41E-08 | 17.08114 |
| rs61914045 | A | G | 0.0309 | 0.2034 | 0.0054 | 7.96E-09 | 10.35978 |
| rs62535714 | A | G | 0.0339 | 0.1639 | 0.0058 | 4.69E-09 | 10.25708 |
| rs66511648 | C | T | 0.0297 | 0.284 | 0.0048 | 6.03E-10 | 15.18706 |
| rs7152906 | C | T | 0.0258 | 0.5196 | 0.0043 | 1.87E-09 | 16.943 |
| rs7241572 | A | G | 0.0323 | 0.2047 | 0.0054 | 2.43E-09 | 10.69502 |
| rs72948506 | A | G | 0.0265 | 0.2975 | 0.0047 | 1.71E-08 | 13.5542 |
| rs7538938 | C | T | 0.0251 | 0.5599 | 0.0043 | 7.29E-09 | 16.86335 |
| rs754287 | A | T | -0.0289 | 0.3664 | 0.0045 | 1.31E-10 | 17.25722 |
| rs7551758 | G | T | 0.0283 | 0.5329 | 0.0043 | 5.11E-11 | 21.39063 |
| rs76954012 | A | T | 0.0412 | 0.0931 | 0.0074 | 2.41E-08 | 17.96952 |
| rs7725715 | A | G | 0.029 | 0.5343 | 0.0043 | 1.61E-11 | 22.5112 |
| rs9364755 | G | A | 0.0283 | 0.2262 | 0.0051 | 3.49E-08 | 11.62374 |
| rs9529218 | T | C | -0.034 | 0.2031 | 0.0054 | 2.23E-10 | 11.79409 |
| rs9536381 | T | C | 0.0255 | 0.3259 | 0.0046 | 2.62E-08 | 14.7386 |

**Table S9. 37 valid IVs used for MR analysis of major depression on KHOA**

| SNPs | Effect  allele | Non-effect  allele | Beta | Effect allele frequency | SE | P-value | F Statistics |
| --- | --- | --- | --- | --- | --- | --- | --- |
| rs1021363 | G | A | -0.03 | 0.6434 | 0.0045 | 2.29E-11 | 20.69073 |
| rs12919291 | C | G | 0.0327 | 0.1884 | 0.0055 | 3.09E-09 | 10.51628 |
| rs12967143 | C | G | -0.0345 | 0.7012 | 0.0047 | 2.53E-13 | 23.57364 |
| rs13037326 | T | C | 0.031 | 0.2597 | 0.0049 | 2.40E-10 | 14.05996 |
| rs1367635 | C | T | 0.0253 | 0.5148 | 0.0043 | 4.35E-09 | 17.2654 |
| rs150186873 | C | A | 0.0704 | 0.0327 | 0.012 | 4.51E-09 | 16.88215 |
| rs150346963 | T | C | 0.0283 | 0.4118 | 0.0044 | 1.16E-10 | 20.07451 |
| rs1931388 | G | A | -0.0295 | 0.4042 | 0.0044 | 1.68E-11 | 22.14638 |
| rs1950829 | G | A | -0.0297 | 0.5173 | 0.0043 | 4.74E-12 | 23.61106 |
| rs198457 | T | C | -0.0315 | 0.1886 | 0.0056 | 1.90E-08 | 18.79061 |
| rs2111592 | A | G | 0.0263 | 0.3141 | 0.0046 | 1.35E-08 | 13.26042 |
| rs2214123 | G | A | -0.0261 | 0.6466 | 0.0045 | 8.56E-09 | 15.37651 |
| rs2418449 | C | T | -0.0281 | 0.281 | 0.0048 | 4.25E-09 | 12.93731 |
| rs247910 | G | A | 0.0237 | 0.457 | 0.0043 | 4.71E-08 | 14.79282 |
| rs2522831 | C | T | 0.024 | 0.4739 | 0.0043 | 2.11E-08 | 15.41759 |
| rs2568958 | A | G | 0.0382 | 0.6042 | 0.0044 | 2.90E-18 | 34.88507 |
| rs28541419 | G | C | -0.0292 | 0.2308 | 0.0052 | 1.76E-08 | 11.07666 |
| rs354155 | C | G | -0.0449 | 0.0923 | 0.0075 | 1.75E-09 | 15.92352 |
| rs3807865 | A | G | 0.031 | 0.4105 | 0.0044 | 1.09E-12 | 24.51364 |
| rs4141983 | C | T | -0.0264 | 0.326 | 0.0046 | 9.69E-09 | 15.41848 |
| rs4497414 | C | T | 0.0291 | 0.44 | 0.0044 | 2.93E-11 | 21.69001 |
| rs4799949 | T | C | -0.0292 | 0.6684 | 0.0046 | 1.40E-10 | 18.75886 |
| rs4936276 | C | G | 0.0278 | 0.622 | 0.0044 | 3.57E-10 | 18.97501 |
| rs508502 | T | C | -0.0264 | 0.2992 | 0.0048 | 3.56E-08 | 11.71557 |
| rs59082935 | T | C | 0.0363 | 0.1342 | 0.0066 | 3.07E-08 | 17.34516 |
| rs59283172 | A | G | -0.039 | 0.1081 | 0.007 | 2.41E-08 | 17.08114 |
| rs61914045 | A | G | 0.0309 | 0.2034 | 0.0054 | 7.96E-09 | 10.35976 |
| rs62535714 | A | G | 0.0339 | 0.1639 | 0.0058 | 4.69E-09 | 10.25707 |
| rs66511648 | C | T | 0.0297 | 0.284 | 0.0048 | 6.03E-10 | 15.18703 |
| rs7152906 | C | T | 0.0258 | 0.5196 | 0.0043 | 1.87E-09 | 16.94297 |
| rs7241572 | A | G | 0.0323 | 0.2047 | 0.0054 | 2.43E-09 | 10.69501 |
| rs7551758 | G | T | 0.0283 | 0.5329 | 0.0043 | 5.11E-11 | 21.39057 |
| rs76954012 | A | T | 0.0412 | 0.0931 | 0.0074 | 2.41E-08 | 17.96951 |
| rs7725715 | A | G | 0.029 | 0.5343 | 0.0043 | 1.61E-11 | 22.51114 |
| rs9364755 | G | A | 0.0283 | 0.2262 | 0.0051 | 3.49E-08 | 11.62372 |
| rs9529218 | T | C | -0.034 | 0.2031 | 0.0054 | 2.23E-10 | 11.79407 |
| rs9536381 | T | C | 0.0255 | 0.3259 | 0.0046 | 2.62E-08 | 14.73857 |

**Table S10. 5 valid IVs used for MR analysis of KOA on major depression**

| SNPs | Effect  allele | Non-effect  allele | Beta | Effect allele frequency | SE | P-value | F Statistics |
| --- | --- | --- | --- | --- | --- | --- | --- |
| rs1078301 | T | A | 0.0679 | 0.2685 | 0.0106 | 1.27E-10 | 15.7266 |
| rs143384 | G | A | -0.0935 | 0.4034 | 0.0095 | 4.77E-23 | 46.92137 |
| rs4775006 | A | C | 0.0578 | 0.4114 | 0.0094 | 8.40E-10 | 18.46292 |
| rs56116847 | A | G | 0.0612 | 0.3563 | 0.0097 | 3.19E-10 | 18.36159 |
| rs8067763 | A | G | -0.0566 | 0.5936 | 0.0095 | 2.39E-09 | 17.12959 |

**Table S11. 18 valid IVs used for MR analysis of HOA on major depression**

| SNPs | Effect  allele | Non-effect  allele | Beta | Effect allele frequency | SE | P-value | F Statistics |
| --- | --- | --- | --- | --- | --- | --- | --- |
| rs10492367 | T | G | 0.1518 | 0.19 | 0.0148 | 1.25E-24 | 35.16401 |
| rs11059094 | T | C | 0.0759 | 0.4777 | 0.0117 | 7.38E-11 | 21.04058 |
| rs115740542 | C | T | 0.1263 | 0.0741 | 0.0224 | 1.60E-08 | 13.04879 |
| rs11583641 | T | C | -0.0811 | 0.2764 | 0.0131 | 5.57E-10 | 13.87777 |
| rs12040949 | T | C | -0.0665 | 0.3843 | 0.012 | 2.83E-08 | 14.59738 |
| rs12209223 | A | C | 0.1558 | 0.1032 | 0.0191 | 3.88E-16 | 16.15675 |
| rs13300602 | G | A | 0.0716 | 0.4508 | 0.0119 | 1.65E-09 | 18.09984 |
| rs1835323 | T | C | -0.0673 | 0.3428 | 0.0123 | 4.56E-08 | 13.09766 |
| rs1913707 | G | A | -0.0795 | 0.3877 | 0.012 | 2.96E-11 | 21.22103 |
| rs2396502 | C | A | 0.0842 | 0.6018 | 0.012 | 2.12E-12 | 23.97529 |
| rs2785988 | A | C | 0.0828 | 0.2988 | 0.0127 | 7.30E-11 | 17.42942 |
| rs2836618 | A | G | 0.0876 | 0.2613 | 0.0132 | 3.20E-11 | 19.72131 |
| rs4338381 | G | A | -0.095 | 0.3681 | 0.0121 | 4.37E-15 | 29.80441 |
| rs7222178 | A | T | 0.0965 | 0.1991 | 0.0146 | 3.77E-11 | 12.13744 |
| rs74767794 | G | A | -0.0751 | 0.3171 | 0.0126 | 2.56E-09 | 15.6686 |
| rs7571789 | C | T | -0.0886 | 0.5239 | 0.0117 | 3.26E-14 | 28.38192 |
| rs798748 | C | T | 0.0715 | 0.6183 | 0.012 | 2.50E-09 | 16.01018 |
| rs80287694 | G | A | 0.1093 | 0.1131 | 0.0184 | 2.66E-09 | 16.96959 |

**Table S12. 21 valid IVs used for MR analysis of KHOA on major depression**

| SNPs | Effect  allele | Non-effect  allele | Beta | Effect allele frequency | SE | P-value | F Statistics |
| --- | --- | --- | --- | --- | --- | --- | --- |
| rs10492367 | T | G | 0.0545 | 0.1895 | 0.0097 | 1.96E-08 | 10.55116 |
| rs10758594 | G | A | 0.0436 | 0.5844 | 0.0077 | 1.69E-08 | 16.02503 |
| rs10948196 | T | A | 0.0426 | 0.3861 | 0.0078 | 4.50E-08 | 13.54532 |
| rs11732213 | C | T | -0.0588 | 0.1945 | 0.0096 | 8.81E-10 | 12.32116 |
| rs11923760 | G | C | -0.0448 | 0.3209 | 0.0082 | 4.16E-08 | 13.73124 |
| rs143384 | G | A | -0.0634 | 0.4035 | 0.0077 | 2.42E-16 | 30.98954 |
| rs17659798 | C | A | -0.0539 | 0.2869 | 0.0085 | 2.06E-10 | 15.766 |
| rs2299285 | A | G | 0.0463 | 0.3434 | 0.008 | 7.57E-09 | 14.5339 |
| rs2472304 | A | G | 0.0452 | 0.668 | 0.0081 | 2.03E-08 | 13.94361 |
| rs2622873 | C | T | -0.0684 | 0.129 | 0.0113 | 1.58E-09 | 18.63215 |
| rs2820443 | C | T | 0.0543 | 0.2985 | 0.0083 | 6.01E-11 | 17.5498 |
| rs2953013 | A | C | -0.0524 | 0.7048 | 0.0083 | 3.07E-10 | 16.83555 |
| rs3821262 | G | A | -0.0554 | 0.474 | 0.0076 | 3.52E-13 | 26.54522 |
| rs4144502 | A | G | 0.0468 | 0.5118 | 0.0076 | 9.48E-10 | 18.83681 |
| rs4630744 | G | A | -0.0538 | 0.4916 | 0.0076 | 2.10E-12 | 25.04691 |
| rs4979341 | T | C | 0.0597 | 0.2697 | 0.0086 | 3.35E-12 | 19.75982 |
| rs56116847 | A | G | 0.0453 | 0.3564 | 0.008 | 1.28E-08 | 15.09042 |
| rs75621460 | A | G | 0.1523 | 0.0267 | 0.0256 | 2.88E-09 | 12.74424 |
| rs7935877 | T | C | -0.0822 | 0.071 | 0.0149 | 3.41E-08 | 12.91869 |
| rs9930333 | G | T | 0.0464 | 0.4234 | 0.0077 | 1.51E-09 | 17.75701 |
| rs9977881 | C | T | 0.0607 | 0.1695 | 0.0102 | 2.54E-09 | 13.71567 |

**Table S13. 91 valid IVs used for MR analysis of schizophrenia on KOA**

| SNPs | Effect  allele | Non-effect  allele | Beta | Effect allele frequency | SE | P-value | F Statistics |
| --- | --- | --- | --- | --- | --- | --- | --- |
| rs4921741 | A | G | -0.056 | 0.7649 | 0.0098 | 1.21E-08 | 10.33072 |
| rs3791710 | T | C | 0.060003 | 0.7964 | 0.0108 | 3.02E-08 | 10.84306 |
| rs10108980 | C | T | -0.0628 | 0.8096 | 0.0106 | 2.73E-09 | 11.10575 |
| rs6520064 | A | G | -0.0585 | 0.799 | 0.0106 | 3.58E-08 | 11.34473 |
| rs1915019 | A | G | 0.057098 | 0.2378 | 0.0098 | 6.57E-09 | 11.34613 |
| rs17194490 | G | T | -0.0782 | 0.8353 | 0.0116 | 1.80E-11 | 11.68412 |
| rs6715366 | G | A | -0.0541 | 0.7224 | 0.0097 | 2.49E-08 | 11.74139 |
| rs2999392 | C | T | -0.0518 | 0.3025 | 0.0094 | 3.05E-08 | 11.76035 |
| rs16867571 | A | G | 0.065703 | 0.7579 | 0.0104 | 2.68E-10 | 12.13121 |
| rs61937595 | C | T | 0.130098 | 0.9057 | 0.0162 | 1.15E-15 | 12.23367 |
| rs62183855 | A | C | 0.066097 | 0.7962 | 0.0111 | 2.66E-09 | 12.2565 |
| rs10861176 | G | A | -0.0555 | 0.2667 | 0.0098 | 1.59E-08 | 13.01154 |
| rs11223774 | A | G | 0.052498 | 0.2913 | 0.0094 | 2.74E-08 | 13.17468 |
| rs11941714 | G | A | 0.051596 | 0.6746 | 0.0093 | 3.07E-08 | 13.23978 |
| rs12771371 | G | A | 0.052403 | 0.6827 | 0.0093 | 1.94E-08 | 13.41093 |
| rs9304548 | C | A | 0.056702 | 0.2599 | 0.01 | 1.59E-08 | 13.452 |
| rs3802924 | A | C | 0.073604 | 0.7995 | 0.0108 | 9.58E-12 | 13.52011 |
| rs6546857 | A | G | -0.0604 | 0.7608 | 0.0102 | 2.74E-09 | 13.57944 |
| rs4653164 | C | T | -0.0511 | 0.3077 | 0.0092 | 3.08E-08 | 13.60881 |
| rs11664298 | G | A | -0.0774 | 0.7901 | 0.0108 | 8.94E-13 | 14.26952 |
| rs1881046 | G | T | 0.050703 | 0.6569 | 0.0092 | 3.39E-08 | 14.36664 |
| rs167924 | A | G | -0.0502 | 0.3778 | 0.009 | 2.34E-08 | 14.3981 |
| rs3739118 | G | A | 0.057004 | 0.712 | 0.0095 | 2.36E-09 | 14.44761 |
| rs62018952 | T | C | -0.0584 | 0.2715 | 0.0097 | 1.94E-09 | 14.70571 |
| rs149165 | T | G | 0.0482 | 0.5314 | 0.0087 | 3.01E-08 | 14.72102 |
| rs6549963 | T | C | 0.048304 | 0.5728 | 0.0088 | 4.31E-08 | 14.76452 |
| rs2333321 | A | G | 0.071204 | 0.209 | 0.0105 | 1.25E-11 | 14.82605 |
| rs2053079 | A | G | -0.0599 | 0.7493 | 0.0101 | 3.01E-09 | 14.85629 |
| rs2381411 | T | C | -0.0504 | 0.5969 | 0.0088 | 1.25E-08 | 14.89494 |
| rs713692 | G | A | -0.0566 | 0.3151 | 0.0095 | 2.67E-09 | 14.99468 |
| rs4575535 | A | G | -0.0558 | 0.308 | 0.0096 | 5.77E-09 | 15.01437 |
| rs4632195 | C | T | -0.0472 | 0.4863 | 0.0086 | 4.59E-08 | 15.03475 |
| rs60135207 | G | T | 0.049599 | 0.5707 | 0.0088 | 1.53E-08 | 15.41558 |
| rs7830315 | T | C | -0.0478 | 0.5025 | 0.0086 | 3.08E-08 | 15.44837 |
| rs2909457 | G | A | 0.049 | 0.466 | 0.0087 | 1.48E-08 | 15.69927 |
| rs2455415 | C | T | -0.04949 | 0.5698 | 0.0088 | 1.69E-08 | 15.71433 |
| rs11136325 | G | A | 0.053797 | 0.3976 | 0.0091 | 3.05E-09 | 15.87048 |
| rs145071536 | T | C | -0.0851 | 0.81 | 0.012 | 1.62E-12 | 15.91213 |
| rs8055219 | G | A | -0.0665 | 0.7749 | 0.0101 | 5.69E-11 | 15.92424 |
| rs13233308 | C | T | 0.048704 | 0.5161 | 0.0086 | 1.75E-08 | 16.01091 |
| rs12652777 | T | C | 0.0488 | 0.4893 | 0.0086 | 1.52E-08 | 16.0982 |
| rs35734242 | T | C | -0.0507 | 0.5727 | 0.0089 | 1.37E-08 | 16.16932 |
| rs217336 | C | A | 0.050303 | 0.5605 | 0.0087 | 8.05E-09 | 16.27975 |
| rs7647398 | C | T | 0.077498 | 0.8008 | 0.0109 | 1.07E-12 | 16.29772 |
| rs500102 | T | C | 0.0517 | 0.4031 | 0.0088 | 4.87E-09 | 16.74919 |
| rs7515363 | C | T | 0.053503 | 0.3863 | 0.0089 | 1.84E-09 | 16.91708 |
| rs132582 | C | T | 0.050997 | 0.441 | 0.0086 | 3.26E-09 | 16.93442 |
| rs215412 | G | A | -0.0577 | 0.6739 | 0.0091 | 2.69E-10 | 17.1435 |
| rs634940 | G | T | -0.0664 | 0.7358 | 0.0099 | 1.78E-11 | 17.20469 |
| rs1901512 | T | C | 0.058401 | 0.3053 | 0.0094 | 5.72E-10 | 17.52865 |
| rs6943762 | T | C | 0.105098 | 0.8722 | 0.0132 | 1.57E-15 | 17.61282 |
| rs11027839 | A | C | -0.0515 | 0.491 | 0.0086 | 2.40E-09 | 17.92637 |
| rs16851048 | T | C | -0.0745 | 0.808 | 0.0107 | 4.15E-12 | 18.05686 |
| rs6974218 | A | C | 0.054895 | 0.6159 | 0.0089 | 6.80E-10 | 18.16636 |
| rs56205728 | G | A | -0.063 | 0.7133 | 0.0097 | 1.01E-10 | 18.60727 |
| rs1430894 | C | T | -0.0533 | 0.5251 | 0.0086 | 6.15E-10 | 19.17167 |
| rs11693094 | C | T | 0.054403 | 0.5341 | 0.0087 | 4.29E-10 | 19.21458 |
| rs12489270 | T | C | -0.0579 | 0.6299 | 0.0089 | 7.47E-11 | 19.22257 |
| rs505061 | C | A | -0.0535 | 0.5148 | 0.0086 | 5.80E-10 | 19.29818 |
| rs10117 | G | A | 0.054999 | 0.6055 | 0.0088 | 4.66E-10 | 19.45996 |
| rs6798742 | A | G | -0.0611 | 0.7034 | 0.0093 | 4.57E-11 | 19.72926 |
| rs2815731 | C | A | 0.060003 | 0.6565 | 0.0091 | 4.39E-11 | 19.87348 |
| rs11534045 | G | A | 0.062796 | 0.6908 | 0.0093 | 1.40E-11 | 20.1094 |
| rs4779050 | T | G | 0.057995 | 0.3705 | 0.0089 | 7.27E-11 | 20.36585 |
| rs56335113 | A | G | 0.064701 | 0.3078 | 0.0094 | 6.02E-12 | 20.55765 |
| rs72802868 | G | T | 0.0692 | 0.7011 | 0.0096 | 4.55E-13 | 21.30576 |
| rs7634476 | A | G | -0.0577 | 0.3985 | 0.0088 | 5.46E-11 | 21.35904 |
| rs2456020 | C | T | 0.081598 | 0.7707 | 0.0102 | 1.13E-15 | 21.39074 |
| rs6538539 | G | T | 0.056796 | 0.4457 | 0.0086 | 4.43E-11 | 21.48843 |
| rs12712510 | T | C | 0.057401 | 0.4623 | 0.0087 | 5.14E-11 | 21.74624 |
| rs11210892 | G | A | 0.0635 | 0.3258 | 0.0091 | 2.68E-12 | 21.95958 |
| rs9876421 | C | T | -0.0625 | 0.6571 | 0.0092 | 9.19E-12 | 22.39806 |
| rs9318627 | A | C | 0.061199 | 0.5922 | 0.0088 | 4.35E-12 | 23.29152 |
| rs6673880 | A | G | -0.0623 | 0.5125 | 0.0091 | 7.20E-12 | 23.39867 |
| rs10035564 | A | G | -0.0668 | 0.6746 | 0.0092 | 4.38E-13 | 23.43298 |
| rs10873538 | T | G | -0.0665 | 0.6601 | 0.0091 | 3.01E-13 | 23.7367 |
| rs12151767 | G | A | 0.061105 | 0.5214 | 0.0086 | 1.31E-12 | 25.24059 |
| rs35351411 | A | C | -0.0635 | 0.4455 | 0.0087 | 2.21E-13 | 26.54408 |
| rs12293670 | A | G | 0.070496 | 0.6651 | 0.0092 | 1.56E-14 | 27.33483 |
| rs2514218 | C | T | 0.070496 | 0.6447 | 0.0092 | 1.35E-14 | 27.33483 |
| rs2252074 | T | G | -0.0685 | 0.6005 | 0.0088 | 6.19E-15 | 27.69991 |
| rs5751191 | T | C | -0.0656 | 0.4953 | 0.0086 | 3.00E-14 | 28.98324 |
| rs4812325 | G | A | -0.0719 | 0.3913 | 0.0089 | 8.96E-16 | 31.43536 |
| rs778371 | A | G | -0.0806 | 0.7203 | 0.0095 | 1.50E-17 | 31.75134 |
| rs1451488 | A | G | -0.07089 | 0.442 | 0.0087 | 4.47E-16 | 32.79353 |
| rs9636107 | A | G | -0.0699 | 0.5309 | 0.0086 | 5.12E-16 | 32.81534 |
| rs4766428 | C | T | -0.075 | 0.5687 | 0.0089 | 3.93E-17 | 35.28152 |
| rs4129585 | A | C | 0.074996 | 0.44 | 0.0087 | 5.11E-18 | 35.64126 |
| rs4702 | G | A | 0.084304 | 0.4431 | 0.0089 | 2.79E-21 | 42.84741 |
| rs2238057 | T | G | -0.0835 | 0.5813 | 0.0087 | 8.50E-22 | 45.7635 |
| rs58120505 | T | C | 0.089603 | 0.5642 | 0.0088 | 2.24E-24 | 49.28458 |

**Table S14. 92 valid IVs used for MR analysis of schizophrenia on HOA**

| SNPs | Effect  allele | Non-effect  allele | Beta | Effect allele frequency | SE | P-value | F Statistics |
| --- | --- | --- | --- | --- | --- | --- | --- |
| rs4921741 | A | G | -0.056 | 0.7658 | 0.0098 | 1.21E-08 | 10.33072 |
| rs3791710 | T | C | 0.060003 | 0.7964 | 0.0108 | 3.02E-08 | 10.84307 |
| rs10108980 | C | T | -0.0628 | 0.8094 | 0.0106 | 2.73E-09 | 11.10576 |
| rs6520064 | A | G | -0.0585 | 0.7997 | 0.0106 | 3.58E-08 | 11.34473 |
| rs1915019 | A | G | 0.057098 | 0.2385 | 0.0098 | 6.57E-09 | 11.34614 |
| rs17194490 | G | T | -0.0782 | 0.8352 | 0.0116 | 1.80E-11 | 11.68412 |
| rs6715366 | G | A | -0.0541 | 0.7226 | 0.0097 | 2.49E-08 | 11.7414 |
| rs2999392 | C | T | -0.0518 | 0.3028 | 0.0094 | 3.05E-08 | 11.76035 |
| rs16867571 | A | G | 0.065703 | 0.7584 | 0.0104 | 2.68E-10 | 12.13122 |
| rs61937595 | C | T | 0.130098 | 0.9056 | 0.0162 | 1.15E-15 | 12.23367 |
| rs62183855 | A | C | 0.066097 | 0.7961 | 0.0111 | 2.66E-09 | 12.25651 |
| rs10861176 | G | A | -0.0555 | 0.2664 | 0.0098 | 1.59E-08 | 13.01155 |
| rs11223774 | A | G | 0.052498 | 0.2917 | 0.0094 | 2.74E-08 | 13.17469 |
| rs11941714 | G | A | 0.051596 | 0.675 | 0.0093 | 3.07E-08 | 13.23978 |
| rs12771371 | G | A | 0.052403 | 0.6826 | 0.0093 | 1.94E-08 | 13.41094 |
| rs9304548 | C | A | 0.056702 | 0.2598 | 0.01 | 1.59E-08 | 13.45201 |
| rs3802924 | A | C | 0.073604 | 0.7989 | 0.0108 | 9.58E-12 | 13.52012 |
| rs6546857 | A | G | -0.0604 | 0.761 | 0.0102 | 2.74E-09 | 13.57945 |
| rs4653164 | C | T | -0.0511 | 0.3079 | 0.0092 | 3.08E-08 | 13.60882 |
| rs11664298 | G | A | -0.0774 | 0.7903 | 0.0108 | 8.94E-13 | 14.26953 |
| rs1881046 | G | T | 0.050703 | 0.6569 | 0.0092 | 3.39E-08 | 14.36665 |
| rs167924 | A | G | -0.0502 | 0.3781 | 0.009 | 2.34E-08 | 14.39811 |
| rs3739118 | G | A | 0.057004 | 0.7121 | 0.0095 | 2.36E-09 | 14.44762 |
| rs62018952 | T | C | -0.0584 | 0.2717 | 0.0097 | 1.94E-09 | 14.70572 |
| rs149165 | T | G | 0.0482 | 0.5316 | 0.0087 | 3.01E-08 | 14.72103 |
| rs6549963 | T | C | 0.048304 | 0.5734 | 0.0088 | 4.31E-08 | 14.76453 |
| rs2333321 | A | G | 0.071204 | 0.2092 | 0.0105 | 1.25E-11 | 14.82606 |
| rs2053079 | A | G | -0.0599 | 0.7492 | 0.0101 | 3.01E-09 | 14.8563 |
| rs2381411 | T | C | -0.0504 | 0.5968 | 0.0088 | 1.25E-08 | 14.89495 |
| rs713692 | G | A | -0.0566 | 0.3156 | 0.0095 | 2.67E-09 | 14.99469 |
| rs4575535 | A | G | -0.0558 | 0.3084 | 0.0096 | 5.77E-09 | 15.01438 |
| rs4632195 | C | T | -0.0472 | 0.486 | 0.0086 | 4.59E-08 | 15.03476 |
| rs60135207 | G | T | 0.049599 | 0.571 | 0.0088 | 1.53E-08 | 15.4156 |
| rs7830315 | T | C | -0.0478 | 0.5021 | 0.0086 | 3.08E-08 | 15.44838 |
| rs2909457 | G | A | 0.049 | 0.4658 | 0.0087 | 1.48E-08 | 15.69928 |
| rs2455415 | C | T | -0.04949 | 0.5702 | 0.0088 | 1.69E-08 | 15.71434 |
| rs11136325 | G | A | 0.053797 | 0.3979 | 0.0091 | 3.05E-09 | 15.87049 |
| rs145071536 | T | C | -0.0851 | 0.8106 | 0.012 | 1.62E-12 | 15.91214 |
| rs8055219 | G | A | -0.0665 | 0.7748 | 0.0101 | 5.69E-11 | 15.92426 |
| rs13233308 | C | T | 0.048704 | 0.5163 | 0.0086 | 1.75E-08 | 16.01092 |
| rs12652777 | T | C | 0.0488 | 0.4888 | 0.0086 | 1.52E-08 | 16.09821 |
| rs35734242 | T | C | -0.0507 | 0.5723 | 0.0089 | 1.37E-08 | 16.16933 |
| rs217336 | C | A | 0.050303 | 0.5608 | 0.0087 | 8.05E-09 | 16.27977 |
| rs7647398 | C | T | 0.077498 | 0.8009 | 0.0109 | 1.07E-12 | 16.29774 |
| rs500102 | T | C | 0.0517 | 0.4032 | 0.0088 | 4.87E-09 | 16.74921 |
| rs7515363 | C | T | 0.053503 | 0.3867 | 0.0089 | 1.84E-09 | 16.9171 |
| rs132582 | C | T | 0.050997 | 0.441 | 0.0086 | 3.26E-09 | 16.93444 |
| rs215412 | G | A | -0.0577 | 0.674 | 0.0091 | 2.69E-10 | 17.14351 |
| rs634940 | G | T | -0.0664 | 0.7352 | 0.0099 | 1.78E-11 | 17.20471 |
| rs1901512 | T | C | 0.058401 | 0.305 | 0.0094 | 5.72E-10 | 17.52866 |
| rs6943762 | T | C | 0.105098 | 0.872 | 0.0132 | 1.57E-15 | 17.61283 |
| rs11027839 | A | C | -0.0515 | 0.4912 | 0.0086 | 2.40E-09 | 17.92638 |
| rs16851048 | T | C | -0.0745 | 0.8081 | 0.0107 | 4.15E-12 | 18.05688 |
| rs6974218 | A | C | 0.054895 | 0.6159 | 0.0089 | 6.80E-10 | 18.16638 |
| rs56205728 | G | A | -0.063 | 0.7127 | 0.0097 | 1.01E-10 | 18.60729 |
| rs1430894 | C | T | -0.0533 | 0.5248 | 0.0086 | 6.15E-10 | 19.17169 |
| rs11693094 | C | T | 0.054403 | 0.5334 | 0.0087 | 4.29E-10 | 19.2146 |
| rs12489270 | T | C | -0.0579 | 0.6296 | 0.0089 | 7.47E-11 | 19.22259 |
| rs505061 | C | A | -0.0535 | 0.5149 | 0.0086 | 5.80E-10 | 19.2982 |
| rs10117 | G | A | 0.054999 | 0.6051 | 0.0088 | 4.66E-10 | 19.45997 |
| rs6798742 | A | G | -0.0611 | 0.7032 | 0.0093 | 4.57E-11 | 19.72929 |
| rs2815731 | C | A | 0.060003 | 0.6564 | 0.0091 | 4.39E-11 | 19.8735 |
| rs11534045 | G | A | 0.062796 | 0.6909 | 0.0093 | 1.40E-11 | 20.10942 |
| rs4779050 | T | G | 0.057995 | 0.3707 | 0.0089 | 7.27E-11 | 20.36587 |
| rs56335113 | A | G | 0.064701 | 0.308 | 0.0094 | 6.02E-12 | 20.55768 |
| rs72802868 | G | T | 0.0692 | 0.7007 | 0.0096 | 4.55E-13 | 21.30579 |
| rs7634476 | A | G | -0.0577 | 0.3982 | 0.0088 | 5.46E-11 | 21.35906 |
| rs2456020 | C | T | 0.081598 | 0.7707 | 0.0102 | 1.13E-15 | 21.39076 |
| rs6538539 | G | T | 0.056796 | 0.4459 | 0.0086 | 4.43E-11 | 21.48845 |
| rs12712510 | T | C | 0.057401 | 0.4626 | 0.0087 | 5.14E-11 | 21.74627 |
| rs11210892 | G | A | 0.0635 | 0.326 | 0.0091 | 2.68E-12 | 21.9596 |
| rs9876421 | C | T | -0.0625 | 0.6567 | 0.0092 | 9.19E-12 | 22.39809 |
| rs9318627 | A | C | 0.061199 | 0.5929 | 0.0088 | 4.35E-12 | 23.29155 |
| rs6673880 | A | G | -0.0623 | 0.5133 | 0.0091 | 7.20E-12 | 23.3987 |
| rs10035564 | A | G | -0.0668 | 0.6742 | 0.0092 | 4.38E-13 | 23.43301 |
| rs10873538 | T | G | -0.0665 | 0.6602 | 0.0091 | 3.01E-13 | 23.73673 |
| rs12151767 | G | A | 0.061105 | 0.5224 | 0.0086 | 1.31E-12 | 25.24062 |
| rs35351411 | A | C | -0.0635 | 0.4453 | 0.0087 | 2.21E-13 | 26.54411 |
| rs12293670 | A | G | 0.070496 | 0.6654 | 0.0092 | 1.56E-14 | 27.33487 |
| rs2514218 | C | T | 0.070496 | 0.6443 | 0.0092 | 1.35E-14 | 27.33487 |
| rs2252074 | T | G | -0.0685 | 0.6003 | 0.0088 | 6.19E-15 | 27.69995 |
| rs5751191 | T | C | -0.0656 | 0.4954 | 0.0086 | 3.00E-14 | 28.98328 |
| rs4812325 | G | A | -0.0719 | 0.392 | 0.0089 | 8.96E-16 | 31.43541 |
| rs778371 | A | G | -0.0806 | 0.7204 | 0.0095 | 1.50E-17 | 31.75139 |
| rs1451488 | A | G | -0.07089 | 0.4424 | 0.0087 | 4.47E-16 | 32.79359 |
| rs9636107 | A | G | -0.0699 | 0.5309 | 0.0086 | 5.12E-16 | 32.8154 |
| rs4766428 | C | T | -0.075 | 0.5685 | 0.0089 | 3.93E-17 | 35.28158 |
| rs4129585 | A | C | 0.074996 | 0.4402 | 0.0087 | 5.11E-18 | 35.64133 |
| rs12129573 | C | A | -0.0778 | 0.6517 | 0.0089 | 2.28E-18 | 36.32373 |
| rs4702 | G | A | 0.084304 | 0.4436 | 0.0089 | 2.79E-21 | 42.84751 |
| rs2238057 | T | G | -0.0835 | 0.5816 | 0.0087 | 8.50E-22 | 45.76362 |
| rs58120505 | T | C | 0.089603 | 0.5639 | 0.0088 | 2.24E-24 | 49.28472 |

**Table S15. 92 valid IVs used for MR analysis of schizophrenia on KHOA**

| SNPs | Effect  allele | Non-effect  allele | Beta | Effect allele frequency | SE | P-value | F Statistics |
| --- | --- | --- | --- | --- | --- | --- | --- |
| rs4921741 | A | G | -0.056 | 0.7649 | 0.0098 | 1.21E-08 | 10.33071 |
| rs3791710 | T | C | 0.060003 | 0.7962 | 0.0108 | 3.02E-08 | 10.84305 |
| rs10108980 | C | T | -0.0628 | 0.8095 | 0.0106 | 2.73E-09 | 11.10574 |
| rs6520064 | A | G | -0.0585 | 0.7994 | 0.0106 | 3.58E-08 | 11.34472 |
| rs1915019 | A | G | 0.057098 | 0.2383 | 0.0098 | 6.57E-09 | 11.34612 |
| rs17194490 | G | T | -0.0782 | 0.8352 | 0.0116 | 1.80E-11 | 11.68411 |
| rs6715366 | G | A | -0.0541 | 0.7224 | 0.0097 | 2.49E-08 | 11.74138 |
| rs2999392 | C | T | -0.0518 | 0.3026 | 0.0094 | 3.05E-08 | 11.76034 |
| rs16867571 | A | G | 0.065703 | 0.7581 | 0.0104 | 2.68E-10 | 12.1312 |
| rs61937595 | C | T | 0.130098 | 0.9057 | 0.0162 | 1.15E-15 | 12.23365 |
| rs62183855 | A | C | 0.066097 | 0.7961 | 0.0111 | 2.66E-09 | 12.25649 |
| rs10861176 | G | A | -0.0555 | 0.2665 | 0.0098 | 1.59E-08 | 13.01153 |
| rs11223774 | A | G | 0.052498 | 0.2914 | 0.0094 | 2.74E-08 | 13.17467 |
| rs11941714 | G | A | 0.051596 | 0.6746 | 0.0093 | 3.07E-08 | 13.23976 |
| rs12771371 | G | A | 0.052403 | 0.6829 | 0.0093 | 1.94E-08 | 13.41091 |
| rs9304548 | C | A | 0.056702 | 0.2599 | 0.01 | 1.59E-08 | 13.45199 |
| rs3802924 | A | C | 0.073604 | 0.7992 | 0.0108 | 9.58E-12 | 13.52009 |
| rs6546857 | A | G | -0.0604 | 0.7609 | 0.0102 | 2.74E-09 | 13.57943 |
| rs4653164 | C | T | -0.0511 | 0.3072 | 0.0092 | 3.08E-08 | 13.6088 |
| rs11664298 | G | A | -0.0774 | 0.7901 | 0.0108 | 8.94E-13 | 14.26951 |
| rs1881046 | G | T | 0.050703 | 0.6568 | 0.0092 | 3.39E-08 | 14.36662 |
| rs167924 | A | G | -0.0502 | 0.3778 | 0.009 | 2.34E-08 | 14.39809 |
| rs3739118 | G | A | 0.057004 | 0.7122 | 0.0095 | 2.36E-09 | 14.44759 |
| rs62018952 | T | C | -0.0584 | 0.2716 | 0.0097 | 1.94E-09 | 14.70569 |
| rs149165 | T | G | 0.0482 | 0.5318 | 0.0087 | 3.01E-08 | 14.721 |
| rs6549963 | T | C | 0.048304 | 0.5728 | 0.0088 | 4.31E-08 | 14.7645 |
| rs2333321 | A | G | 0.071204 | 0.2088 | 0.0105 | 1.25E-11 | 14.82603 |
| rs2053079 | A | G | -0.0599 | 0.7494 | 0.0101 | 3.01E-09 | 14.85627 |
| rs2381411 | T | C | -0.0504 | 0.597 | 0.0088 | 1.25E-08 | 14.89493 |
| rs713692 | G | A | -0.0566 | 0.3156 | 0.0095 | 2.67E-09 | 14.99466 |
| rs4575535 | A | G | -0.0558 | 0.308 | 0.0096 | 5.77E-09 | 15.01435 |
| rs4632195 | C | T | -0.0472 | 0.4861 | 0.0086 | 4.59E-08 | 15.03473 |
| rs60135207 | G | T | 0.049599 | 0.5709 | 0.0088 | 1.53E-08 | 15.41557 |
| rs7830315 | T | C | -0.0478 | 0.5024 | 0.0086 | 3.08E-08 | 15.44835 |
| rs2909457 | G | A | 0.049 | 0.466 | 0.0087 | 1.48E-08 | 15.69925 |
| rs2455415 | C | T | -0.04949 | 0.5699 | 0.0088 | 1.69E-08 | 15.71431 |
| rs11136325 | G | A | 0.053797 | 0.3976 | 0.0091 | 3.05E-09 | 15.87046 |
| rs145071536 | T | C | -0.0851 | 0.8101 | 0.012 | 1.62E-12 | 15.91211 |
| rs8055219 | G | A | -0.0665 | 0.775 | 0.0101 | 5.69E-11 | 15.92422 |
| rs13233308 | C | T | 0.048704 | 0.5162 | 0.0086 | 1.75E-08 | 16.01089 |
| rs12652777 | T | C | 0.0488 | 0.4891 | 0.0086 | 1.52E-08 | 16.09818 |
| rs35734242 | T | C | -0.0507 | 0.5726 | 0.0089 | 1.37E-08 | 16.1693 |
| rs217336 | C | A | 0.050303 | 0.5606 | 0.0087 | 8.05E-09 | 16.27973 |
| rs7647398 | C | T | 0.077498 | 0.8008 | 0.0109 | 1.07E-12 | 16.2977 |
| rs500102 | T | C | 0.0517 | 0.4034 | 0.0088 | 4.87E-09 | 16.74917 |
| rs7515363 | C | T | 0.053503 | 0.3863 | 0.0089 | 1.84E-09 | 16.91706 |
| rs132582 | C | T | 0.050997 | 0.4407 | 0.0086 | 3.26E-09 | 16.9344 |
| rs215412 | G | A | -0.0577 | 0.6738 | 0.0091 | 2.69E-10 | 17.14347 |
| rs634940 | G | T | -0.0664 | 0.7355 | 0.0099 | 1.78E-11 | 17.20467 |
| rs1901512 | T | C | 0.058401 | 0.305 | 0.0094 | 5.72E-10 | 17.52862 |
| rs6943762 | T | C | 0.105098 | 0.8722 | 0.0132 | 1.57E-15 | 17.61279 |
| rs11027839 | A | C | -0.0515 | 0.4908 | 0.0086 | 2.40E-09 | 17.92634 |
| rs16851048 | T | C | -0.0745 | 0.8078 | 0.0107 | 4.15E-12 | 18.05684 |
| rs6974218 | A | C | 0.054895 | 0.6156 | 0.0089 | 6.80E-10 | 18.16634 |
| rs56205728 | G | A | -0.063 | 0.713 | 0.0097 | 1.01E-10 | 18.60725 |
| rs1430894 | C | T | -0.0533 | 0.525 | 0.0086 | 6.15E-10 | 19.17164 |
| rs11693094 | C | T | 0.054403 | 0.5339 | 0.0087 | 4.29E-10 | 19.21456 |
| rs12489270 | T | C | -0.0579 | 0.6298 | 0.0089 | 7.47E-11 | 19.22254 |
| rs505061 | C | A | -0.0535 | 0.5152 | 0.0086 | 5.80E-10 | 19.29815 |
| rs10117 | G | A | 0.054999 | 0.6054 | 0.0088 | 4.66E-10 | 19.45993 |
| rs6798742 | A | G | -0.0611 | 0.7034 | 0.0093 | 4.57E-11 | 19.72923 |
| rs2815731 | C | A | 0.060003 | 0.6564 | 0.0091 | 4.39E-11 | 19.87345 |
| rs11534045 | G | A | 0.062796 | 0.6907 | 0.0093 | 1.40E-11 | 20.10937 |
| rs4779050 | T | G | 0.057995 | 0.3705 | 0.0089 | 7.27E-11 | 20.36582 |
| rs56335113 | A | G | 0.064701 | 0.3081 | 0.0094 | 6.02E-12 | 20.55762 |
| rs72802868 | G | T | 0.0692 | 0.7008 | 0.0096 | 4.55E-13 | 21.30573 |
| rs7634476 | A | G | -0.0577 | 0.3985 | 0.0088 | 5.46E-11 | 21.359 |
| rs2456020 | C | T | 0.081598 | 0.7708 | 0.0102 | 1.13E-15 | 21.3907 |
| rs6538539 | G | T | 0.056796 | 0.4455 | 0.0086 | 4.43E-11 | 21.48839 |
| rs12712510 | T | C | 0.057401 | 0.4623 | 0.0087 | 5.14E-11 | 21.7462 |
| rs11210892 | G | A | 0.0635 | 0.3256 | 0.0091 | 2.68E-12 | 21.95954 |
| rs9876421 | C | T | -0.0625 | 0.6567 | 0.0092 | 9.19E-12 | 22.39802 |
| rs9318627 | A | C | 0.061199 | 0.5924 | 0.0088 | 4.35E-12 | 23.29148 |
| rs6673880 | A | G | -0.0623 | 0.5128 | 0.0091 | 7.20E-12 | 23.39863 |
| rs10035564 | A | G | -0.0668 | 0.6746 | 0.0092 | 4.38E-13 | 23.43294 |
| rs10873538 | T | G | -0.0665 | 0.6598 | 0.0091 | 3.01E-13 | 23.73666 |
| rs12151767 | G | A | 0.061105 | 0.5216 | 0.0086 | 1.31E-12 | 25.24054 |
| rs35351411 | A | C | -0.0635 | 0.4453 | 0.0087 | 2.21E-13 | 26.54402 |
| rs12293670 | A | G | 0.070496 | 0.6651 | 0.0092 | 1.56E-14 | 27.33477 |
| rs2514218 | C | T | 0.070496 | 0.6449 | 0.0092 | 1.35E-14 | 27.33477 |
| rs2252074 | T | G | -0.0685 | 0.6003 | 0.0088 | 6.19E-15 | 27.69985 |
| rs5751191 | T | C | -0.0656 | 0.4952 | 0.0086 | 3.00E-14 | 28.98317 |
| rs4812325 | G | A | -0.0719 | 0.3914 | 0.0089 | 8.96E-16 | 31.43528 |
| rs778371 | A | G | -0.0806 | 0.7203 | 0.0095 | 1.50E-17 | 31.75126 |
| rs1451488 | A | G | -0.07089 | 0.4421 | 0.0087 | 4.47E-16 | 32.79345 |
| rs9636107 | A | G | -0.0699 | 0.5309 | 0.0086 | 5.12E-16 | 32.81525 |
| rs4766428 | C | T | -0.075 | 0.5687 | 0.0089 | 3.93E-17 | 35.28142 |
| rs4129585 | A | C | 0.074996 | 0.4401 | 0.0087 | 5.11E-18 | 35.64116 |
| rs12129573 | C | A | -0.0778 | 0.652 | 0.0089 | 2.28E-18 | 36.32355 |
| rs4702 | G | A | 0.084304 | 0.4437 | 0.0089 | 2.79E-21 | 42.84726 |
| rs2238057 | T | G | -0.0835 | 0.5814 | 0.0087 | 8.50E-22 | 45.76333 |
| rs58120505 | T | C | 0.089603 | 0.5645 | 0.0088 | 2.24E-24 | 49.28438 |

**Table S16. 5 valid IVs used for MR analysis of KOA on schizophrenia**

| SNPs | Effect  allele | Non-effect  allele | Beta | Effect allele frequency | SE | P-value | F Statistics |
| --- | --- | --- | --- | --- | --- | --- | --- |
| rs143384 | G | A | -0.0935 | 0.4034 | 0.0095 | 4.77E-23 | 48.0238 |
| rs4775006 | A | C | 0.0578 | 0.4114 | 0.0094 | 8.40E-10 | 18.89668 |
| rs56116847 | A | G | 0.0612 | 0.3563 | 0.0097 | 3.19E-10 | 18.79297 |
| rs8067763 | A | G | -0.0566 | 0.5936 | 0.0095 | 2.39E-09 | 17.53202 |
| rs9277552 | T | C | -0.064 | 0.2105 | 0.0114 | 1.97E-08 | 11.51613 |

**Table S17. 15 valid IVs used for MR analysis of HOA on schizophrenia**

| SNPs | Effect  allele | Non-effect  allele | Beta | Effect allele frequency | SE | P-value | F Statistics |
| --- | --- | --- | --- | --- | --- | --- | --- |
| rs1835323 | T | C | -0.0673 | 0.3428 | 0.0123 | 4.56E-08 | 13.09766 |
| rs11583641 | T | C | -0.0811 | 0.2764 | 0.0131 | 5.57E-10 | 13.87777 |
| rs12040949 | T | C | -0.0665 | 0.3843 | 0.012 | 2.83E-08 | 14.59738 |
| rs74767794 | G | A | -0.0751 | 0.3171 | 0.0126 | 2.56E-09 | 15.6686 |
| rs798748 | C | T | 0.0715 | 0.6183 | 0.012 | 2.50E-09 | 16.01018 |
| rs12209223 | A | C | 0.1558 | 0.1032 | 0.0191 | 3.88E-16 | 16.15675 |
| rs2785988 | A | C | 0.0828 | 0.2988 | 0.0127 | 7.30E-11 | 17.42942 |
| rs13300602 | G | A | 0.0716 | 0.4508 | 0.0119 | 1.65E-09 | 18.09984 |
| rs2836618 | A | G | 0.0876 | 0.2613 | 0.0132 | 3.20E-11 | 19.72131 |
| rs11059094 | T | C | 0.0759 | 0.4777 | 0.0117 | 7.38E-11 | 21.04058 |
| rs1913707 | G | A | -0.0795 | 0.3877 | 0.012 | 2.96E-11 | 21.22103 |
| rs2396502 | C | A | 0.0842 | 0.6018 | 0.012 | 2.12E-12 | 23.97529 |
| rs7571789 | C | T | -0.0886 | 0.5239 | 0.0117 | 3.26E-14 | 28.38192 |
| rs4338381 | G | A | -0.095 | 0.3681 | 0.0121 | 4.37E-15 | 29.80441 |
| rs10492367 | T | G | 0.1518 | 0.19 | 0.0148 | 1.25E-24 | 35.16401 |

**Table S18. 16 valid IVs used for MR analysis of KHOA on schizophrenia**

| SNPs | Effect  allele | Non-effect  allele | Beta | Effect allele frequency | SE | P-value | F Statistics |
| --- | --- | --- | --- | --- | --- | --- | --- |
| rs11732213 | C | T | -0.0588 | 0.1945 | 0.0096 | 8.81E-10 | 10.71471 |
| rs2299285 | A | G | 0.0463 | 0.3434 | 0.008 | 7.57E-09 | 11.90904 |
| rs10492367 | T | G | 0.0545 | 0.1895 | 0.0097 | 1.96E-08 | 12.4357 |
| rs17659798 | C | A | -0.0539 | 0.2869 | 0.0085 | 2.06E-10 | 12.57488 |
| rs2472304 | A | G | 0.0452 | 0.668 | 0.0081 | 2.03E-08 | 13.11437 |
| rs4630744 | G | A | -0.0538 | 0.4916 | 0.0076 | 2.10E-12 | 13.14987 |
| rs9930333 | G | T | 0.0464 | 0.4234 | 0.0077 | 1.51E-09 | 13.16823 |
| rs4144502 | A | G | 0.0468 | 0.5118 | 0.0076 | 9.48E-10 | 13.33715 |
| rs2622873 | C | T | -0.0684 | 0.129 | 0.0113 | 1.58E-09 | 14.11234 |
| rs2953013 | A | C | -0.0524 | 0.7048 | 0.0083 | 3.07E-10 | 14.84905 |
| rs9277552 | T | C | -0.0592 | 0.2098 | 0.0093 | 2.37E-10 | 15.89545 |
| rs3821262 | G | A | -0.0554 | 0.474 | 0.0076 | 3.52E-13 | 18.3971 |
| rs9977881 | C | T | 0.0607 | 0.1695 | 0.0102 | 2.54E-09 | 19.10043 |
| rs4979341 | T | C | 0.0597 | 0.2697 | 0.0086 | 3.35E-12 | 20.47114 |
| rs75621460 | A | G | 0.1523 | 0.0267 | 0.0256 | 2.88E-09 | 22.04735 |
| rs143384 | G | A | -0.0634 | 0.4035 | 0.0077 | 2.42E-16 | 25.15506 |
